# (+)/(−)-Phaeocaulin A-D, four pairs of new enantiomeric germacrane-type sesquiterpenes from *Curcuma phaeocaulis* as natural nitric oxide inhibitors

**DOI:** 10.1038/srep43576

**Published:** 2017-03-08

**Authors:** Gui-yang Xia, De-juan Sun, Jiang-hao Ma, Yue Liu, Feng Zhao, Paul Owusu Donkor, Li-qin Ding, Li-xia Chen, Feng Qiu

**Affiliations:** 1 School of Chinese Materia Medica and Tianjin State Key Laboratory of Modern Chinese Medicine, Tianjin University of Traditional Chinese Medicine, 312 Anshanxi Road, Nankai District, Tianjin 300193, People’s Republic of China; 2 Department of Natural Products Chemistry, School of Traditional Chinese Materia Medica, Key Laboratory of Structure-Based Drug Design & Discovery, Ministry of Education, Shenyang Pharmaceutical University, Shenyang 110016, People’s Republic of China; 3 School of Pharmacy, Key Laboratory of Molecular Pharmacology and Drug Evaluation (Yantai University), Ministry of Education, Collaborative Innovation Center of Advanced Drug Delivery System and Biotech Drugs in Universities of Shandong, Yantai University, Yantai, 264005, People’s Republic of China

## Abstract

Germacrane-type sesquiterpenes, with a flexible 10-membered ring unit as the structural and conformational features, play a central role in the biosynthesis and synthesis of other sesquiterpenes. In this report, two pairs of new sesquiterpene alkaloids, (+)/(−)-phaeocaulin A [(+)-**1**/(−)-**1**] and B [(+)-**2**/(−)-**2**], and two pairs of new sesquiterpenes, (+)/(−)-phaeocaulin C [(+)-**3**/(−)-**3**] and D [(+)-**4**/(−)-**4**], along with one related known analog (**5**), were isolated from the rhizomes of *Curcuma phaeocaulis*. The absolute configurations of (+)-**1**/(−)-**1**, (+)-**2**/(−)-**2**, (+)-**3**/(−)-**3** and (+)-**4**/(−)-**4** were unambiguously determined by analysis of single-crystal X-ray diffractions and quantum chemical electronic circular dichroism (ECD) method. It is noteworthy that (+)/(−)-phaeocaulin A [(+)-**1**/(−)-**1**] and B [(+)-**2**/(−)-**2**] are two pairs of rare N-containing germacrane-type sesquiterpenes. A possible biogenetic pathway for **1–5** was postulated. All of the isolated compounds were tested for their inhibitory activity against LPS-induced nitric oxide production in RAW 264.7 macrophages.

Natural products and their derivatives have been a rich source of bioactive compounds for drug discovery and development^1^. To date, more than 200 different sesquiterpene skeletons have been discovered, and these are predominantly formed from farnesyl diphosphate (FDP) as a common acyclic precursor by enzymatic cyclizations and further transformations^2^,^3^. Germacrane-type sesquiterpenes, with unique structural and conformational features, are naturally occurring in plants, bacteria, fungi, and marine invertebrates. Owing to their central role in the biosynthesis of other sesquiterpenes and their potent bioactivities, germacrane-type sesquiterpenes have stimulated efforts in their isolation, synthesis and structural modification for drug discovery^4^,^5^,^6^,^7^. It is not uncommon for natural products to have one or more stereogenic centers with a significant influence on biological activity^8^,^9^,^10^. Different single enantiomers may have different pharmacokinetic properties (absorption, distribution, biotransformation, and excretion) and quantitatively or qualitatively different pharmacologic or toxicologic effects^11^. The separation and configurational assignment of optically pure compounds are an important yet challenging process in structure elucidation.

*Curcuma phaeocaulis* Valeton, belonging to the family Zingiberaceae, is widely distributed in the southern regions of the People’s Republic of China such as Sichuan, Yunnan, Guangdong, and Fujian provinces. The rhizomes of this plant, known as *Rhizoma curcumae (Ezhu* in Chinese), are an important crude drug frequently listed in prescriptions of traditional Chinese medicine (TCM) for the treatment of Blood Stasis Syndrome (BSS) caused by the obstruction of blood circulation, such as arthralgia, psychataxia, and dysmenorrhea^12^,^13^. Recent phytochemical investigations of this plant have revealed that its main constituents are sesquiterpenoids^13^,^14^, and these constituents exhibit anti-inflammatory^15^, antitumor^16^,^17^, and platelet aggregation inhibitory^18^ activities. As part of our continuing investigations into biologically active sesquiterpenoids from *C. phaeocaulis*, and to provide a potential explanation for its usage of treating inflammatory diseases in China, the remaining fractions were further fractionated to afford two rare N-containing germacrane-type sesquiterpenes, two new germacrane-type sesquiterpenes (Fig. 1), and one known germacrane derivative. Although germacrane-type sesquiterpenes are not very rare, the discovery of the unusual N atom in the skeleton of germacrane-type sesquiterpenes, coupled with the existence of enantiomers in compounds **1–5** for the single chiral center at C-8 is especially interesting. Optically pure enantiomers (+**)-1/(−)-1**, (+**)-2/(−)-2**, (+**)-3/(−)-3**, and (+**)-4/(−)-4** were obtained with the help of chiral high-performance liquid chromatography (HPLC) separation. We describe, herein, the isolation and unequivocal characterization of these compounds, as well as their inhibitory effects on nitric oxide (NO) production in lipopolysaccharide (LPS)-activated macrophages. To our knowledge, this represents the first instance of enantiomeric separation of germacrane-type sesquiterpenes by chiral HPLC column that enables us to obtain optically pure materials for further investigations.

## Results

### Structure elucidation

**Figure 2:**
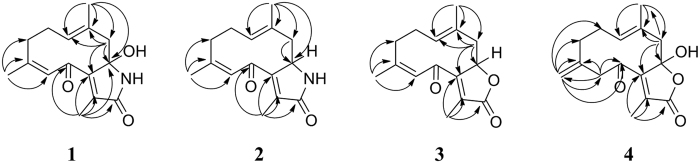
Key HMBC correlations of compounds 1, 2, 3 and 4.

Phaeocaulin A (**1**) was obtained as white needles from methanol. It was assigned the molecular formula C_15_H_19_NO_3_ (seven degrees of unsaturation) on the basis of HRESIMS analysis. The IR spectrum exhibited absorptions at 3384 and 1634 cm^−1^ which were typical for the lactam group^13^. The ^1^H NMR spectroscopic data (Table 1) exhibited signals corresponding to three olefinic methyl groups [(*δ*_H_ 1.66, 1.88 and 1.93 (each 3H, s)] and two olefinic protons [*δ*_H_ 6.37 (1H, s) and 4.92 (1H, dd, *J* = 10.6, 3.6)]. The ^13^C NMR data (Table 1) indicated the presence of two carbonyl carbons (*δ*_C_ 172.1 and 195.3), six olefinic carbons (*δ*_C_ 128.4, 130.9, 139.4, 140.3, 148.7, and 153.7), and three methyl carbons (*δ*_C_ 10.1, 18.8, and 25.2). Extensive comparison of the ^1^H and ^13^C NMR data of **1** with those of (1*E*,4*Z*)-8-hydroxy-6-oxogermacra-1(10),4,7(11)-trieno-12,8-lactone (**5**) which was obtained from *Chloranthus henryi*^19^, suggested that the structure of **1** resembled that of **5**, except for the low-frequency shift of C-8. This appearance could be explained if C-8 was attached to a less electronegative atom than an oxygen atom, for instance, a nitrogen atom. This inference was confirmed by the HMBC correlations from H_3_–15 to C-1/C-8/C-9/C-10, H_2_-9 to C-1/C-7/C-8/C-10/C-15, and H_3_-13 to C-6/C-7/C-8/C-11/C-12 (Fig. 2). It was reported that the ^13^C NMR method could be used to predict the configuration of trisubstituted double bonds containing one methyl substituent. If the resonance for the vinylic methyl group appears at a value greater than 20 ppm, the double bond has a (*Z*)-configuration, whereas if the value is less than 20 ppm, an (*E*)-configuration is present^20^. Therefore, the 1,10- and 4,5-double bonds were assigned as (*E*)- and (*Z*)-configurations, respectively, due to the chemical shifts of C-15 (*δ*_C_ 18.8) and C-14 (*δ*_C_ 25.2). On the basis of the above evidence, the structure of **1** was established as (1*E*,4*Z*)-8-hydroxy-6-oxogermacra-1(10),4,7(11)-trieno-12,8-lactam.

Phaeocaulin B (**2**) was obtained as white needles from methanol and has the molecular formula C_15_H_19_NO_2_ with seven degrees of unsaturation, as deduced from the HRESIMS analysis (*m/z* 268.1307 [M+Na]^+^, calcd for 268.1308). The structure of **2** was mainly determined by comparing its NMR spectroscopic data (Table 1) with those of **1**. The absence of the hydroxy group at C-8 in **2** was suggested by the molecular formula (C_15_H_19_NO_2_) and the up-field shift of C-8 (*δ*_C_ 60.4 in **2** and *δ*_C_ 92.8 in **1**), as well as the presence of the hydrogen signal at *δ*_H_ 4.64. This deduction was supported by the HMBC correlations from H-9a to C-8, and H_3_-15 to C-1/C-7/C-8/C-9/C-10. Thus, the structure of compound **2** was established as (1*E*,4*Z*)-6-oxogermacra-1(10),4,7(11)-trieno-12,8-lactam.

Phaeocaulin C (**3**) was obtained as colorless cube crystals from methanol and yielded a quasi-molecular ion peak in the HRESIMS spectrum at *m/z* 247.1336 [M+H]^+^, which indicated a molecular formula of C_15_H_18_O_3_ in conjunction with ^13^C NMR data. A single-crystal X-ray diffraction analysis of **3** (See Supplementary data) showed that the main skeleton of **3** was a germacrane-type sesquiterpene. The IR spectrum revealed the presence of an *α*,*β*-unsaturated *γ*-lactone (1762 cm^−1^) group. In the ^1^H NMR spectrum of **3** (Table 1), the characteristic protons for two olefinic protons [*δ*_H_ 5.01 (1H, brs) and 6.17 (1H, s)] and three methyl groups [*δ*_H_ 1.60 (3H, s), 1.93 (3H, s), and 2.03 (3H, s)] were observed. The ^13^C NMR spectroscopic data of **3** (Table 1) showed the presence of 15 carbon atoms, including three methyls, three methylenes, three methines and six quaternary carbons. Comparing the ^1^H and ^13^C NMR spectra of **3** with those of **5**, the hemiketal carbon present in **5**^19^ was missing and an additional oxymethine was observed at *δ*_H_ 5.24 (1H, brs) and *δ*_C_ 80.1. According to the aforementioned information, the structure of **3** was assigned as an 8-deoxy derivative of **5**. This deduction was supported by the HMBC correlations from H-9a to C-1/C-7/C-8/C-10/C-15. Thus, the structure of compound **3** was assigned as (1*E*,4*Z*)-6-oxogermacra-1(10),4,7(11)-trieno-12,8-lactone.

Phaeocaulin D (**4**) was assigned the molecular formula C_15_H_18_O_4_ according to a quasi-molecular ion at *m/z* 285.1096 [M+Na]^+^ in its HRESIMS spectrum. The ^1^H NMR spectrum showed signals corresponding to two methyl groups *δ*_H_ 1.64 (3H, s) and 2.00 (3H, s). The ^13^C NMR spectroscopic data (Table 1) indicated the presence of two carbonyl carbons (*δ*_C_ 197.1 and 170.4), one hemiketal carbon (*δ*_C_ 110.4), six olefinic carbons (*δ*_C_ 119.2, 130.5, 130.7, 135.4, 140.3 and 154.7), and two methyl carbons (*δ*_C_ 10.6 and 17.8). The ^1^H and ^13^C NMR spectra of **4** were similar to those of **5**^19^, except for the absence of one methyl group and the appearance of one 1,1-disubstituted double bond. Its position could be determined by the key HMBC correlations from H-14a to C-3/C-4/C-5/C-6, H-14b to C-2/C-3/C-4/C-5/C-6, and H_2_-5 to C-3/C-4/C-6/C-14. The substituted positions of the CH_3_-13 and CH_3_-15 were determined by the HMBC correlations from H_3_-13 to C-7/C-11/C-12 and H_3_-15 to C-1/C-9/C-10. The 1,10-double bond was assigned an (*E*)-configuration due to the chemical shift of C-15 (*δ*_C_ 17.8)^20^. On the basis of all the above evidences, the structure of **4** was elucidated as (1*E*)-8-hydroxy-6-oxogermacra-1(10),4(14),7(11)-trieno-12,8-lactone.

### Stereochemical issues

Although compounds **1**–**5** all possess a chiral carbon at C-8, the specific optical rotations were, in all cases, close to zero. Moreover, no Cotton effects (CEs) were observed in their ECD spectra. It suggested that these chiral compounds might be obtained as racemic mixtures. This speculation was confirmed by the X-ray analysis of **1–3** and **5** which crystallized in space groups containing inversion centers or glide planes^21^.

**Figure 3:**
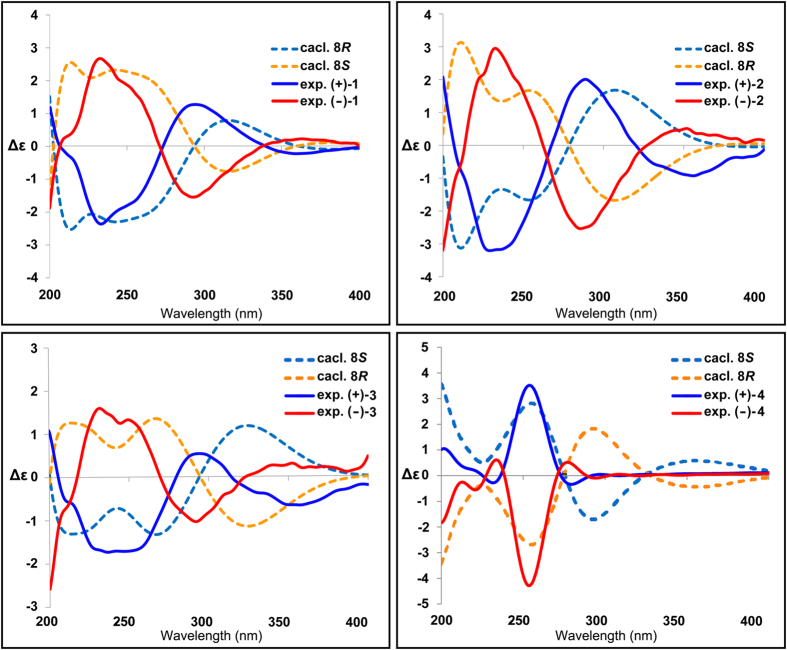
Experimental ECD spectra of compounds (+)-1/(−)-1, (+)-2/(−)-2, (+)-3/(−)-3 and (+)-4/(−)-4 and calculated ECD spectra of *8S/8R* of 1–4.

Compounds **1–5** were further subjected to HPLC separation on chiral columns (Chiralpak IE and Chiralpak AD-RH). The chiral HPLC analysis of each of **1**-**4** showed well-resolved peaks of two enantiomers on the Chiralpak IE column (250 mm × 4.6 mm, 5 *μ*m; Daicel) with *n*-hexane/isopropanol at a rate of 0.8 mL min^−1^. The relative abundance of each pair was ca. 1:1 according to their relative peak areas in the HPLC chromatograms. Efforts were made to get the enantiomers of **5** separated. Unfortunately, no well-resolved peaks were observed with changing columns and methods. Finally, chiral semi-preparative HPLC purifications were undertaken for compounds **1–4**, yielding (+)-**1**/(−)-**1**, (+)-**2**/(−)-**2**, (+)-**3**/(−)-**3**, and (+)-**4**/(−)-**4**. Each of these compounds showed typical antipodal ECD curves (Fig. 3) and specific rotations of opposite sign.

**Figure 4:**
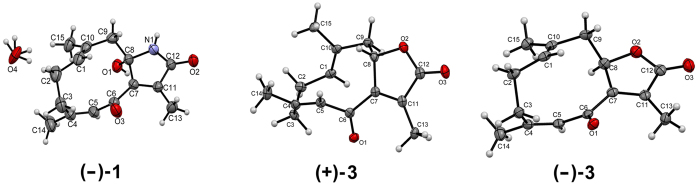
ORTEP drawings of compounds (−)-1, (+)-3, and (−)-3.

Pure enantiomers of (−)-**1** (CCDC 1486406), (+)-**3** (CCDC 1486411), and (−)-**3** (CCDC 1486410) were further recrystallized in methanol to obtain single crystals for X-ray structure determination using Cu Kα radiation (Fig. 4) which allowed for the determination of the absolute configuration of C-8 in (−)-**1** and (+)-**3** as 8*S* and that of their enantiomers (+)-**1** and (−)-**3** as 8*R*.

Computational calculations of ECD^22^,^23^,^24^ spectra of the 8*S*/8*R* enantiomers of compounds **1**–4 were performed. Comparison of these calculated spectra with the experimental ECD spectra obtained from the isolated enantiomers allowed us to determine their absolute configurations (Fig. 3). Consequently, the absolute configurations of C-8 in (+**)-1/(−)-1**, (+**)-2/(−)-2**, (+**)-3/(−)-3** and (+**)-4/(−)-4** were unambiguously determined.

### Inhibitory effect of the isolated compounds on NO production induced by LPS in macrophages

Nitric oxide produced by a group of nitric oxide synthases (NOSs) is highly diffusible across cell membranes and modifies many biological molecules. It plays an important role in the inflammatory process, and an inhibitor of NO production may be considered as a potential anti-inflammatory agent^25^,^26^. To confirm the bioactive secondary metabolites responsible for the anti-inflammatory activity of *C. phaeocaulis*, all isolated compounds were tested for their inhibitory effects on NO production induced by LPS in macrophages (pure enantiomers of **4** were not measured due to paucity of the sample) (Table 2). In comparison with the positive control, hydrocortisone (IC_50_ 48.66 μM), most of the compounds exhibited moderate inhibitory activities against NO production with IC_50_ values in the range of 17.34 to 30.02 μM. The possible mechanisms of these active compounds remain to be further explored.

## Discussion

The characteristic features for each pair of enantiomers were summarized in Table 3. The empirical CD rules have been successfully employed in determining the stereochemistry of the *α*,*β*-unsaturated lactone or lactam rings in various natural products^27^,^28^,^29^. However, it seems that the rules are not entirely applicable to these four pairs of germacrane-type sesquiterpenes, which could be attributed to the neighboring high conformational flexible ten-membered ring and the presence of the polyunsaturated conjugated chromophores around the stereogenic center (C-8)^28^,^29^. This was confirmed by the shifts of CEs at ca. 220 and 250 nm in the CD spectra of (−)-**4**/(+)-**4** when compared to (−)-**1**/(+)-**1**, (−)-**2**/(+)-**2**, and (−)-**3**/(+)-**3**. Here, characteristic ECD spectra of four pairs of unambiguously determined germacrane-type sesquiterpene enantiomers were provided. Further study is also required to elucidate the underlying mechanism behind the observation of the CEs.

**Figure 5:**
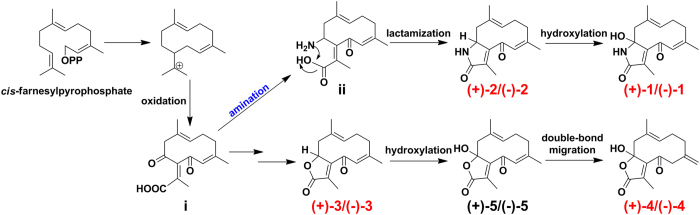
Plausible biogenetic pathway of 1–5.

The germacrane-type sesquiterpenes with a flexible 10-membered ring system, are biogenetically generated from FDP and play a central role in the biosynthesis or synthesis of other sesquiterpenes. The discovery of sesquiterpene alkaloids (+)-**1/**(−)-**1** and (+)-**2/**(−)-**2** in *Curcuma* genus is rather unusual from a chemotaxonomic perspective. These type of alkaloids are synthesized primarily from non-amino acid precursors, with the nitrogen atom being inserted into the structure at a relatively late stage by amination processes^30^. The proposed biosynthesis of **1–5** was shown in Fig. 5. First, the 10-membered ring systems could be formed by a cyclization of the *cis*-farnesylpyrophosphate. Following a series of enzymatic oxidations, the intermediate **i** could be generated. Then, the important intermediate **ii** could be derived from **i** via amination reactions. The racemization of **1**–**5** could be explained by the intramolecular lactamization or lactonization, which might be non-stereoselective. Under enzyme catalysis, (+)-**2/**(−)-**2** and (+)-**3/**(−)-**3** could then undergo hydroxylation to yield the racemates (+)-**1/**(−)-**1** and (+)-**5/**(−)-**5**, respectively. Ultimately, the racemates (+)-**4/**(−)-**4** could be generated from (+)-**5/**(−)-**5** via the migration of the double bonds.

## Conclusion

The current study reported the isolation and structure elucidation of two rare germacrane-type sesquiterpene alkaloids (**1** and **2**) and two new germacrane-type sesquiterpenes (**3** and **4**) from the rhizomes of *Curcuma phaeocaulis*. The chiral resolution of the enantiomeric germacrane-type sesquiterpenes, (+)-**1**/(−)-**1**, (+)-**2**/(−)-**2**, (+)-**3**/(−)-**3**, and (+)-**4**/(−)-**4**, permitted the unambiguous definition of the absolute configurations of the optically pure enantiomers via X-ray diffraction analysis and computation of ECD spectra, which provides powerful models for the absolute configuration studies of this class of compounds. Inhibitory effects of the isolated compounds on nitric oxide production in LPS-activated macrophages were evaluated. Most of the isolated compounds exhibited more potent inhibition than the positive control, hydrocortisone, indicating their potential as promising compounds for further research and development of anti-inflammatory agents. The whole spectroscopic data, including the computation of ECD spectra, will provide additional evidence for the absolute configuration of similar structures.

## Methods

### General

The melting point (uncorrected) was determined on an X-4 digital display micromelting point apparatus. Optical rotations were measured with a Perkin-Elmer 241 polarimeter. UV spectra were recorded on a Shimadzu UV 2201 spectrophotometer. IR spectra were recorded on a Bruker IFS 55 spectrometer. CD spectra were recorded on a Bio-Logic Science MOS-450 spectrometer. NMR experiments were performed on Bruker ARX-300 and AV-600 spectrometers. HRESIMS were obtained on an Agilent 6210 TOF mass spectrometer. Silica gel GF254 prepared for TLC and silica gel (200–300 mesh) for column chromatography were obtained from Qingdao Marine Chemical Factory (Qingdao, People’s Republic of China). Octadecyl silica gel was purchased from Merck Chemical Company Ltd. RP-HPLC separations were conducted using an LC-6AD liquid chromatograph with a YMC Pack ODS-A column (250 × 20 mm, 5 μm, 120 Å) and an SPD-10A VP UV/VIS detector. Analysis and chiral purifications of racemates of **1–4** were carried out on a Chiralpak IE column (150 mm × 4.6 mm, 5 μm; Daicel Chemical Industries, Ltd). All reagents were of HPLC or analytical grade and were purchased from Tianjin Damao Chemical Company. Spots were detected on TLC plates under UV light or by heating after spraying with anisaldehyde-H_2_SO_4_.

### Plant material

Rhizomes of *C. phaeocaulis* were collected from Chengdu, Sichuan Province, China, and identified by Professor Qishi Sun, Department of Pharmaceutical Botany, School of Traditional Chinese Materia Medica, Shenyang Pharmaceutical University. A voucher specimen (CP-20100715) has been deposited in the herbarium of the Department of Natural Products Chemistry, Shenyang Pharmaceutical University.

### Extraction and isolation

Dry rhizomes of *C. phaeocaulis* (10 kg) were cut into approximately 2 cm pieces and extracted with 95% aqueous EtOH (2 × 100L × 2 h). After evaporation of the combined EtOH extracts *in vacuo*, the resulting concentrated extract (0.6 kg) was suspended in H_2_O (3L), and partitioned successively with cyclohexane, EtOAc, and *n*-BuOH (3 × 3L). The cyclohexane extract (170 g) was subjected to silica gel column (10 × 80 cm) eluted with hexane/EtOAc (100:1, 40:1, 20:1, 10:1, 4:1, 2:1, 1:1, and 0:1 v/v) to obtain fractions CA–CH. Fraction CA (5 g) was subjected to silica gel column (3 × 40 cm) eluted with petroleum ether/acetone (from 40:1 to 0:1) to produce seven fractions (CA1–CA7). Fraction CB (20 g) was subjected to silica gel column (6 × 80 cm) eluted with cyclohexane/acetone (from 40:1 to 0:1) to produce five fractions (CB1–CB5). Fraction CB1 (2 g) was chromatographed over a silica gel column (3 × 40 cm) eluted with petroleum ether/acetone (from 40:1 to 0:1) to produce fractions CB11–CB13. Fraction CB11 (20.0 mg) was recrystallized to give compound **1** (22.6 mg), while (+)-**1** (3.9 mg, *t*_R_ = 7.0 min) and (−)-**1** (3.8 mg, *t*_R_ = 15.4 min) were obtained from a chiral column (Chiralpak IE) under normal phase conditions (*n*-hexane:isopropanol = 1:1) at 0.8 mL/min using a UV detector at 220 nm. Fraction CB4 (3.5 g) was subjected to reversed-phase C_18_ silica gel column (2.5 × 30 cm) eluted with MeOH/H_2_O (1:9 to 8:2) to yield CB41, CB42 and CB43. CB42 (80 mg) was separated by HPLC (60% MeOH/H_2_O) to afford compound **2** (10.5 mg, *t*_R_ = 45 min), while (+)-**2** (2.7 mg, *t*_R_ = 9.7 min) and (−)-**2** (2.5 mg, *t*_R_ = 10.9 min) were obtained from a chiral column (Chiralpak IE) under normal phase conditions (*n*-hexane:isopropanol = 1:1) at 0.8 mL/min using a UV detector at 220 nm. Fraction CC (19 g) was subjected to silica gel column (6 × 80 cm) chromatography eluting with petroleum ether/EtOAc (from 40:1 to 0:1) to produce seven fractions (CC1–CC7). Fraction CC1 (25 mg) was recrystallized to give compound **5** (14.2 mg). Fraction CD (6.2 g) was chromatographed using reversed-phase C_18_ silica gel column (2.5 × 30 cm) eluting with MeOH/H_2_O (30:70, 50:50, 70:30 and 100:0, v/v) to give three fractions CD1–CD3, and subfraction CD1 (54 mg) was separated by preparative HPLC (50% MeOH/H_2_O, 6 mL/min) to afford compound **4** (2.5 mg, *t*_R_ = 60 min), while (+)-**4** (0.6 mg, *t*_R_ = 9.7 min) and (−)-**4** (0.6 mg, *t*_R_ = 11.1 min) were obtained from a chiral column (Chiralpak IE) under normal phase conditions (*n*-hexane:isopropanol = 4:1) at 0.8 mL/min using a UV detector at 220 nm. The EtOAc extract (105 g) was subjected to silica gel column (10 × 80 cm) eluted with cyclohexane/acetone (100:1, 40:1, 20:1, 10:1, 4:1, 2:1, 1:1, and 0:1 v/v) to obtain five fractions (EA–EF). Fraction EC (15 g) was subjected to silica gel column (6 × 80 cm) eluted with a gradient of increasing acetone (0–100%) in *n*-hexane to afford fractions EC1–EC7. EC3 (4.8 g) was chromatographed over a reversed-phase C_18_ silica gel column (2.5 × 30 cm) eluted with MeOH/H_2_O (30:70, 50:50, 70:30 and 100:0 v/v) to give four fractions EC3-1 to EC3-4, and subfraction EC3-3 (160 mg) was separated by preparative HPLC (40% MeOH/H_2_O, 6.0 mL/min) to afford compound **3** (33.2 mg, *t*_R_ = 73 min), while (+)-**3** (4.3 mg, *t*_R_ = 10.8 min) and (−)-**3** (4.2 mg, *t*_R_ = 12.2 min) were obtained from a chiral column (Chiralpak IE) under normal phase conditions (*n*-hexane:isopropanol = 1:1) at 0.8 mL/min using a UV detector at 220 nm.

### Spectroscopic data of the isolated compounds

**Phaeocaulin A (1):** white needles; 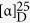 −4.5 (*c* 0.1, MeOH); UV (MeOH) λ_max_ (log ε) 233 (4.14) nm; IR (KBr) *v*_*max*_: 3384, 2945, 2833, 1634, 1450, 1384, 1118, 1030 cm^−1^; HR-ESI-MS *m/z* 284.1257 [M+Na]^+^ (calcd for C_15_H_19_NO_3_Na, 284.1263); ^1^H and ^13^C NMR data, see Table 1.

(+)-**Phaeocaulin A** [(+)-**1]:** white powder; 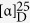
+25.7 (c 0.05, MeOH); CD (CH_3_OH, 1.9 mM) λ_max_ (Δ*ε*) 233 (−5.70), 294 (+3.03), 360 (−0.56).

(−)-**Phaeocaulin A** [(−)-**1]:** colorless needles; m.p. 179.0–180.0 °C; 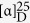 −32.9 (c 0.05, MeOH); CD (CH_3_OH, 1.5 mM) λ_max_ (Δ*ε*) 233 (+2.66), 293 (−1.56), 363 (+0.22).

**Phaeocaulin B (2):** white needles; 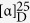
+5.0 (c 0.1, MeOH); UV (MeOH) λ_max_ (log ε) 242 (3.90) nm; IR (KBr) *v*_*max*_: 3407, 2936, 2833, 1692, 1660, 1631, 1448, 1384, 1118, 1030 cm^−1^; HR-ESI-MS *m/z* 268.1307 [M+Na]^+^ (calcd for C_15_H_19_NO_2_Na, 268.1308); ^1^H and ^13^C NMR data, see Table 1.

(+)-**Phaeocaulin B [(**+)-**2]:** white powder; 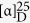
+43.1 (c 0.05, MeOH); CD (CH_3_OH, 0.4 mM) λ_max_ (Δ*ε*) 229 (−3.21), 288 (+2.01), 356 (−0.93).

(−)-**Phaeocaulin B [**(−)-**2]:** white powder; 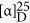 −32.0 (c 0.05, MeOH); CD (CH_3_OH, 0.4 mM) λ_max_ (Δ*ε*) 233 (+2.95), 286 (−2.54), 352 (+0.51).

**Phaeocaulin C (3):** colorless cube crystals; 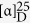 −2.4 (*c* 0.07, MeOH); UV (MeOH) λ_max_ (log *ε*) 228 (4.02) nm; IR (KBr) *v*_max_: 2930, 2855, 1762, 1667, 1644, 1622, 1444, 1384, 1154, 1015 cm^−1^; HRESIMS (positive) *m/z*: 247.1336 [M+H]^+^ (calcd for C_15_H_19_O_3_, 247.1334); ^1^H and ^13^C NMR data, see Table 1.

(+**)-Phaeocaulin C [(**+)-**3]:** colorless needles; m.p. 108.0–109.0 °C; 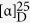
+68.0 (c 0.04, MeOH); CD (CH_3_OH, 0.4 mM) λ_max_ (Δ*ε*) 237 (−1.74), 295 (+0.53), 357 (−0.67).

(−)**-Phaeocaulin C [**(−)-**3]:** colorless needles; m.p. 108.0–109.0 °C; 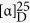 −46.3 (c 0.04, MeOH); CD (CH_3_OH, 0.4 mM) λ_max_ (Δ*ε*) 232 (+1.59), 292 (−1.04), 353 (+0.29).

**Phaeocaulin D (4):** yellowish oil; 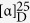
+6.0 (c 0.1, MeOH); UV (MeOH) λ_max_ (log *ε*) 213 (1.63) nm; IR (KBr) *v*_max_: 3398, 2943, 2833, 1764, 1642, 1449, 1384, 1127, 1030 cm^−1^; HRESIMS (positive) *m/z*: 285.1096 [M+Na]^+^ (calcd for C_15_H_18_O_4_Na, 285.1097); ^1^H and ^13^C NMR data, see Table 1.

(+**)-Phaeocaulin D [(**+)-**4]:** yellowish oil; 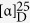
+106.7 (c 0.015, MeOH); CD (CH_3_OH, 0.6 mM) λ_max_ 254 (+1.86).

(−)**-Phaeocaulin D [**(−)-**4]:** yellowish oil; 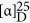 −88.9 (c 0.02, MeOH); CD (CH_3_OH, 0.8 mM) λ_max_ 254 (−1.70).

Single-Crystal X-ray Diffraction Analysis and Crystallographic Data of Compounds **1**, (−)-**1**, **2**, **3**, (+)-**3**, (−)-**3**, and **5**, NO production bioassay, see Supplementary information.

## Additional Information

**How to cite this article**: Xia, G.- *et al*. (+)/(-)-Phaeocaulin A-D, four pairs of new enantiomeric germacrane-type sesquiterpenes from *Curcuma phaeocaulis* as natural nitric oxide inhibitors. *Sci. Rep.* **7**, 43576; doi: 10.1038/srep43576 (2017).

**Publisher's note:** Springer Nature remains neutral with regard to jurisdictional claims in published maps and institutional affiliations.

## Supplementary Material

Supplementary Information

Supplementary Dataset 1

Supplementary Dataset 2

Supplementary Dataset 3

Supplementary Dataset 4

Supplementary Dataset 5

Supplementary Dataset 6

Supplementary Dataset 7

## Figures and Tables

**Figure 1:**
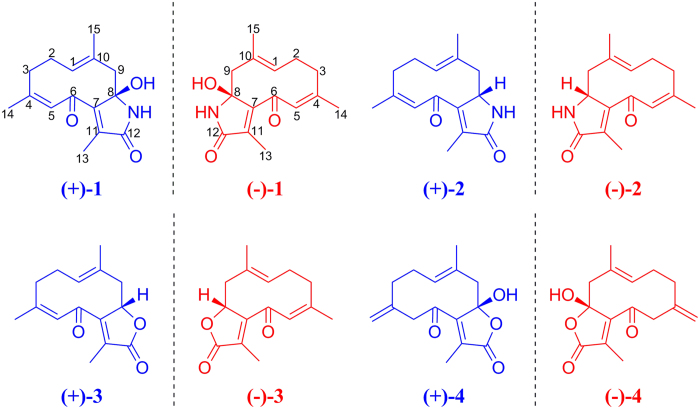
Chemical structures of (+)-1/(−)-1, (+)-2/(−)-2, (+)-3/(−)-3 and (+)-4/(−)-4.

**Table 1:** ^1^H NMR (600 MHz) and ^13^C NMR data for compounds 1–4 (*δ* in ppm, *J* in Hz).

Position	1^a^		2^a^		3^b^		4^b^	
	^1^H	^13^C^c^	^1^H	^13^C^c^	^1^H	^13^C^d^	^1^H	^13^C^d^
1	4.92 (dd, 10.6, 3.6)	128.4	4.90 (m)	129.2	5.01 (brs)	128.7	5.27 (d, 7.9)	130.7
2	2.06 (m)	26.9	2.16 (m)	28.4	2.16 (m)	27.0	2.14 (m)	29.3
	2.20 (ddd, 24.0, 12.0, 3.3)		2.16 (m)		2.16 (m)		2.22 (m)	
3	2.89 (m)	31.0	3.00 (m)	32.6	3.01 (m)	30.8	2.40 (m)	36.2
	2.11 (m)		2.10 (m)		2.14 (m)		2.12 (m)	
4		148.7		151.4		150.6		140.3
5	6.37 (s)	130.9	6.30 (s)	130.6	6.17 (brs)	127.7	3.45 (s)	50.7
6		195.3		196.0		191.1		197.1
7		153.7		156.3		158.1		154.7
8		92.8	4.64 (d, 9.8)	60.4	5.24 (brs)	80.1		110.4
9	2.24 (d, 12.8)	50.3	2.63 (dd, 11.8, 3.1)	47.3	2.86 (dd, 11.8, 3.7)	45.5	2.32 (d, 13.3)	49.9
	2.69 (d, 12.8)		1.96 (m)		2.18 (m)		2.86 (d, 13.3)	
10		139.4		138.1		133.6		135.4
11		140.3		140.9		132.9		130.5
12		172.1		175.1		172.9		170.4
13	1.93 (s)	10.1	1.91 (s)	10.8	2.03 (s)	9.9	2.00 (s)	10.6
14	1.88 (s)	25.2	1.92 (s)	25.9	1.93 (s)	25.0	4.95 (s)	119.2
							5.12 (s)	
15	1.66 (s)	18.8	1.61 (s)	17.7	1.60 (s)	16.6	1.64 (s)	17.8

^a^Spectra were obtained in CD_3_OD.

^b^Spectra were obtained in CDCl_3_.

^c^Recorded at 75 MHz.

^d^Recorded at 150 MHz.

**Table 2:** NO inhibitory activity of compounds 1–5.

Compounds	IC_50_ ± SD(μM)	Compounds	IC_50_ ± SD(μM)
(±)-**1**	57.63 ± 4.44	(±)-**3**	28.72 ± 2.80
(+)-**1**	35.97 ± 3.13	(+)-**3**	20.43 ± 2.26
(−)-**1**	57.25 ± 4.27	(−)-**3**	23.85 ± 2.20
(±)-**2**	44.18 ± 3.91	(±)-**4**	29.17 ± 2.78
(+)-**2**	24.02 ± 1.99	(±)-**5**	17.34 ± 1.65
(−)-**2**	47.95 ± 3.68	**Hydrocortisone**	48.66 ± 3.26

**Table 3:** Characteristic features for the enantiomers.

		(+)-1	(−)-1	(+)-2	(−)-2	(+)-3	(−)-3	(+)-4	(−)-4
C-8		*R*	*S*	*S*	*R*	*S*	*R*	*S*	*R*
Retention time (min)^a^		7.0	15.4	9.7	10.9	10.8	12.2	9.7	11.1
Optical rotation 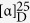		+25.7	−32.9	+43.1	−32.0	+68.0	−46.3	+106.7	−88.9
CEs	200–205 nm	+	−	+	−	+	−	+	−
	220–260 nm	−	+	−	+	−	+	+	−
	280–310 nm	+	−	+	−	+	−	−	+

^a^For chromatographic conditions, see extraction and isolation section.
