# Supplementary material for: (+)/(−)-Phaeocaulin A-D, four pairs of new enantiomeric germacrane-type sesquiterpenes from Curcuma phaeocaulis as natural nitric oxide inhibitors
Source: Sci Rep. 2017 Mar 8;7:43576. doi: 10.1038/srep43576 (PMC5341095; doi:10.1038/srep43576)
Supplement: Supplementary Information [file srep43576-s1.pdf]

Supplementary information

(+)/(−)-Phaeocaulin A-D, four pairs of new enantiomeric germacrane-type sesquiterpenes from *Curcuma phaeocaulis* as natural nitric oxide inhibitors

Gui-yang Xia[1,2], De-juan Sun[2], Jiang-hao Ma[2], Yue Liu[2], Feng Zhao[3], Paul Owusu Donkor[1], Li-qin Ding[1], Li-xia Chen[*,2] & Feng Qiu[*,1]

[1]School of Chinese Materia Medica and Tianjin State Key Laboratory of Modern Chinese Medicine, Tianjin University of Traditional Chinese Medicine, 312 Anshanxi Road, Nankai District, Tianjin 300193, People's Republic of China. [2]Department of Natural Products Chemistry, School of Traditional Chinese Materia Medica, Key Laboratory of Structure-Based Drug Design & Discovery, Ministry of Education, Shenyang Pharmaceutical University, Shenyang 110016, People's Republic of China. [3]School of Pharmacy, Key Laboratory of Molecular Pharmacology and Drug Evaluation (Yantai University), Ministry of Education, Collaborative Innovation Center of Advanced Drug Delivery System and Biotech Drugs in Universities of Shandong, Yantai University, Yantai, 264005, People's Republic of China.

Correspondence and requests for materials should be addressed to F. Q. (fengqiu20070118@163.com) or L.-X. C. (syzyclx@163.com).

Contents of Supporting Information

Figure S1. ^{1}H NMR (600 MHz) spectrum of compound **1** in CD_3OD. 1
Figure S2. ^{1}H NMR (600 MHz) spectrum of compound **(+)-1** in CD_3OD. 2
Figure S3. ^{1}H NMR (600 MHz) spectrum of compound **(-)-1** in CD_3OD. 3
Figure S4. ^{13}C NMR (75 MHz) spectrum of compound **1** in CD_3OD. 4
Figure S5. ^{13}C NMR (150 MHz) spectrum of compound **(+)-1** in CD_3OD. 5
Figure S6. ^{13}C NMR (150 MHz) spectrum of compound **(-)-1** in CD_3OD. 6
Figure S7. HSQC spectrum of compound **1** in CD_3OD. 7
Figure S8. HMBC spectrum of compound **1** in CD_3OD. 8
Figure S9. IR spectrum of compound **1**. 9
Figure S10. HRESIMS data of compound **1**. 10
Figure S11. UV spectrum of compound **1**. 10
Figure S12. ^{1}H NMR (600 MHz) spectrum of compound **2** in CD_3OD. 11
Figure S13. ^{13}C NMR (75 MHz) spectrum of compound **2** in CD_3OD. 12
Figure S14. HSQC spectrum of compound **2** in CD_3OD. 13
Figure S15. HMBC spectrum of compound **2** in CD_3OD. 14
Figure S16. IR spectrum of compound **2**. 15
Figure S17. HRESIMS data of compound **2**. 16
Figure S18. UV spectrum of compound **2**. 16
Figure S19. ^{1}H NMR (600 MHz) spectrum of compound **3** in $CDCl_3$. 1
Figure S20. ^{1}H NMR (600 MHz) spectrum of compound **(+)-3** in $CDCl_3$. 2
Figure S21. ^{1}H NMR (600 MHz) spectrum of compound **(-)-3** in $CDCl_3$. 3
Figure S22. ^{13}C NMR (150 MHz) spectrum of compound **3** in $CDCl_3$. 4
Figure S23. ^{13}C NMR (150 MHz) spectrum of compound **(+)-3** in $CDCl_3$. 5
Figure S24. ^{13}C NMR (150 MHz) spectrum of compound **(-)-3** in $CDCl_3$. 6
Figure S25. HSQC spectrum of compound **3** in $CDCl_3$. 7
Figure S26. HMBC spectrum of compound **3** in $CDCl_3$. 8
Figure S27. IR spectrum of compound **3**. 9
Figure S28. HRESIMS data of compound **3**. 10
Figure S29. UV spectrum of compound **3**. 11
Figure S30. ^{1}H NMR (600 MHz) spectrum of compound **4** in $CDCl_3$. 12
Figure S31. ^{13}C NMR (150 MHz) spectrum of compound **4** in $CDCl_3$. 13
Figure S32. HSQC spectrum of compound **4** in $CDCl_3$. 14
Figure S33. HMBC spectrum of compound **4** in $CDCl_3$. 15
Figure S34. IR spectrum of compound **4**. 16
Figure S35. HRESIMS data of compound **4**. 17
Figure S36. UV spectrum of compound **4**. 17
Single-Crystal X-ray Diffraction Analysis and Crystallographic Data of Compounds 1, (-)-1, 2, 3, (+)-3, (-)-3, and 5. 18
Quantum chemical ECD calculation of 1-4 20

NO production bioassay 21
References... 21

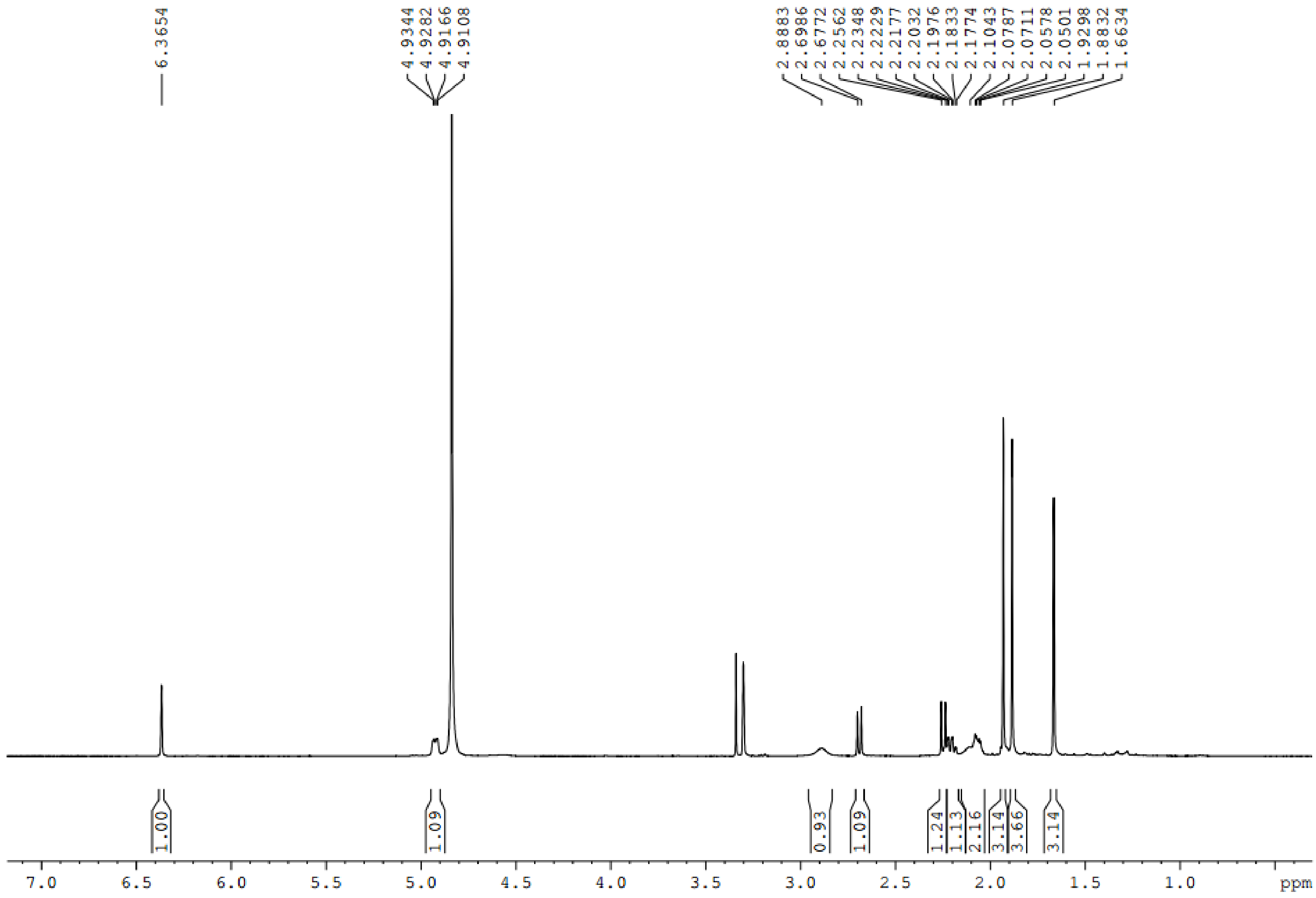

Figure S1. ^{1}H NMR (600 MHz) spectrum of compound **1** in CD_3OD

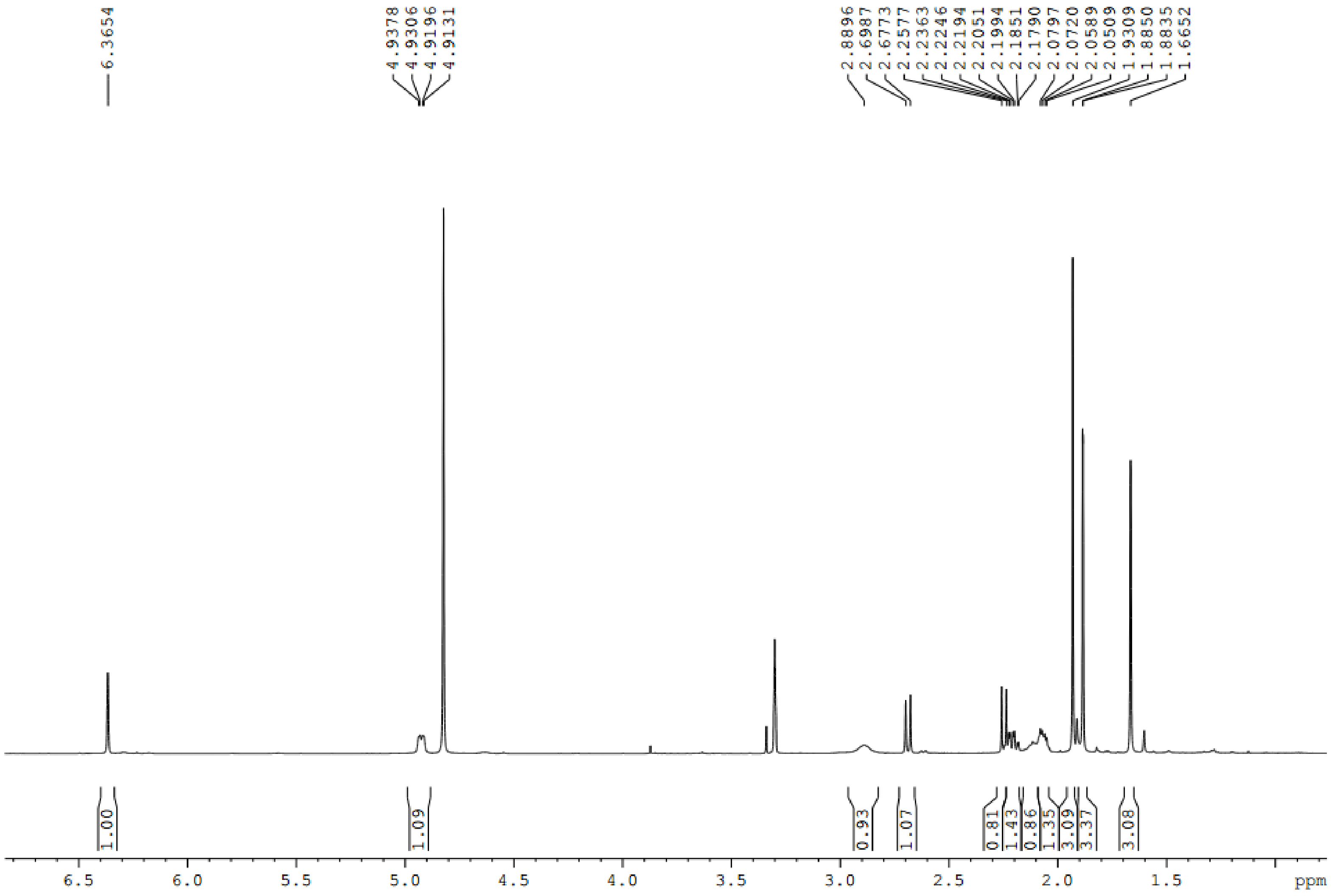

Figure S2. ^{1}H NMR (600 MHz) spectrum of compound **(+)-1** in CD_3OD

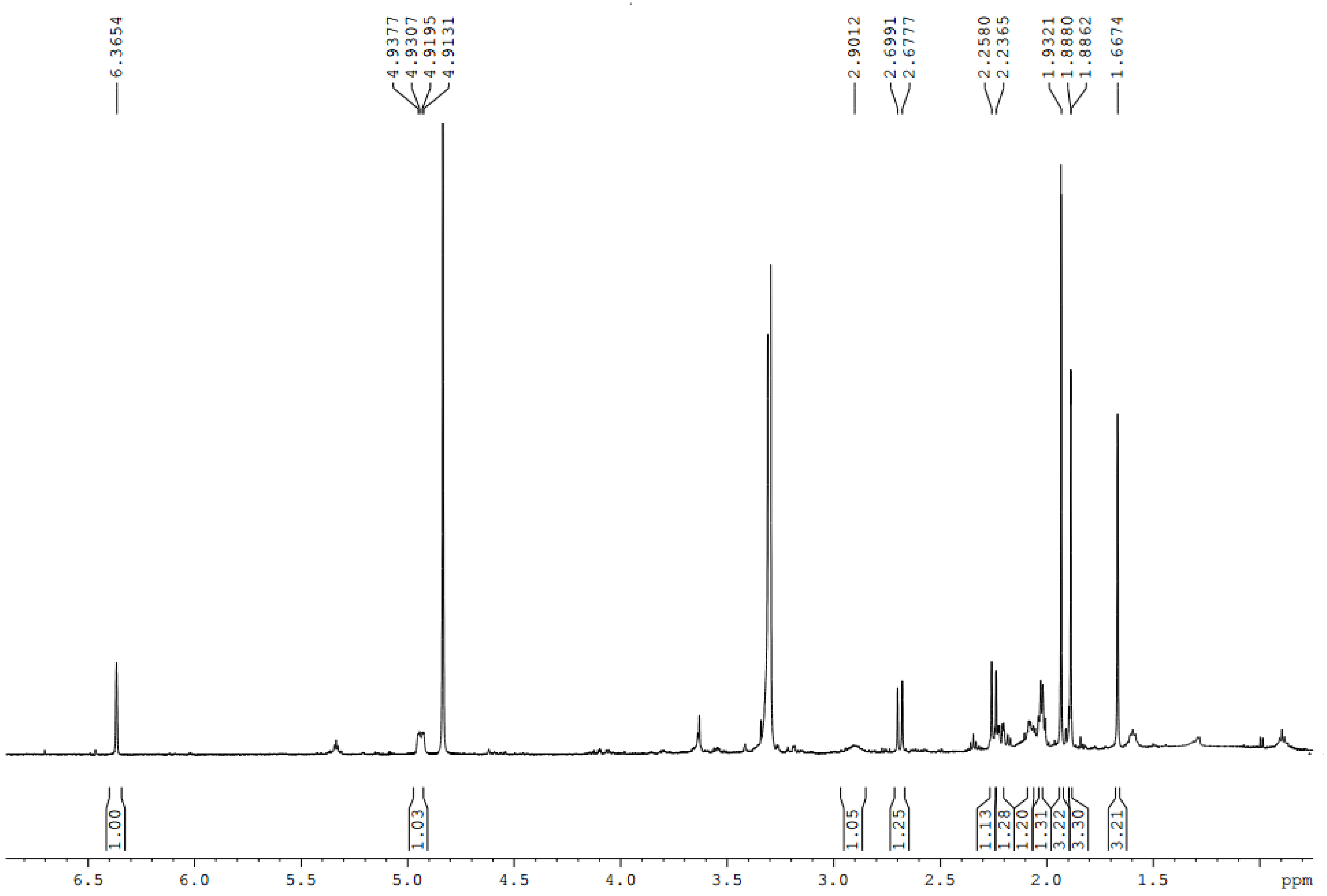

Figure S3. ^{1}H NMR (600 MHz) spectrum of compound **(-)-1** in CD_3OD

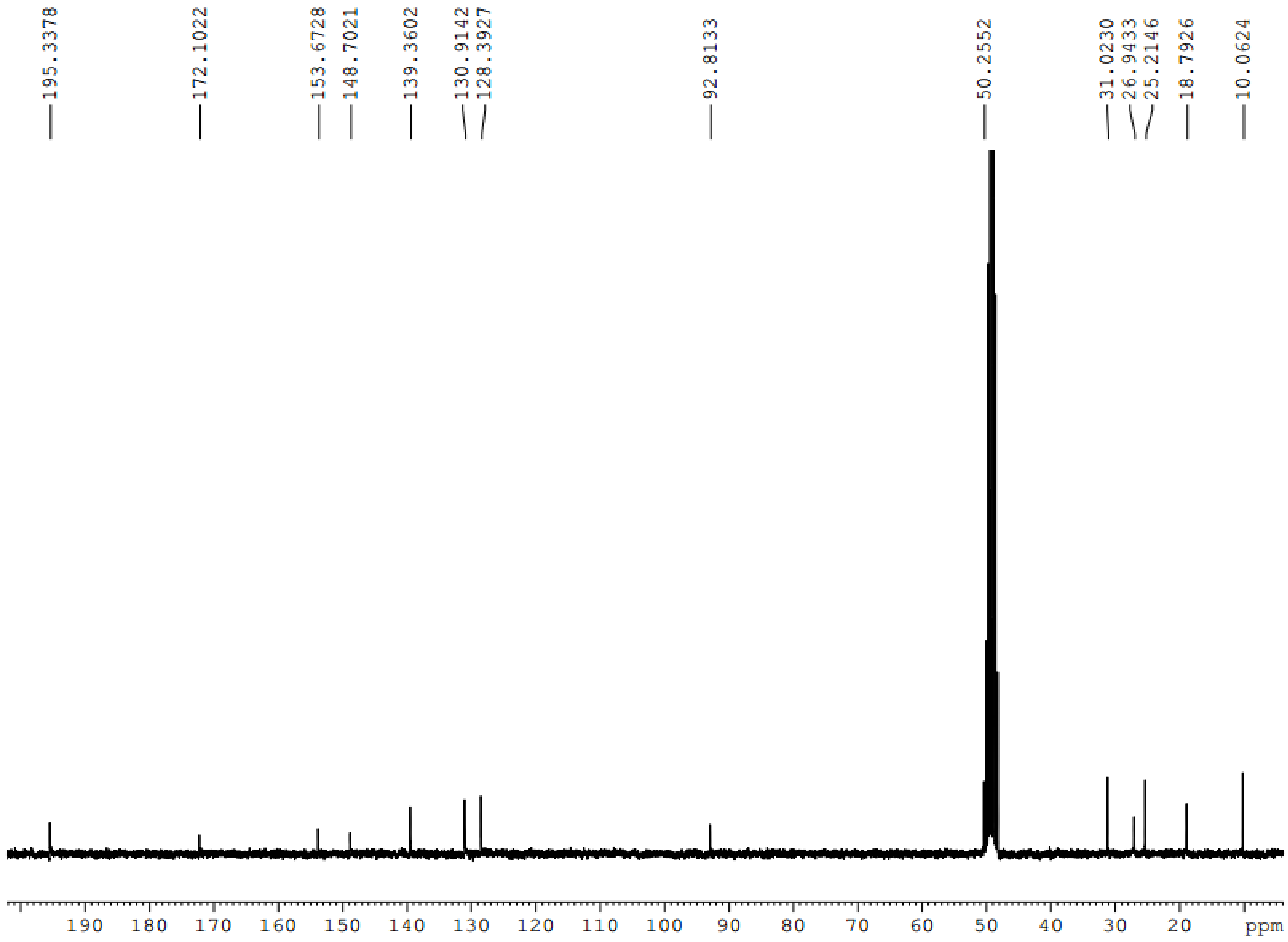

Figure S4. ^{13}C NMR (75 MHz) spectrum of compound **1** in CD_3OD

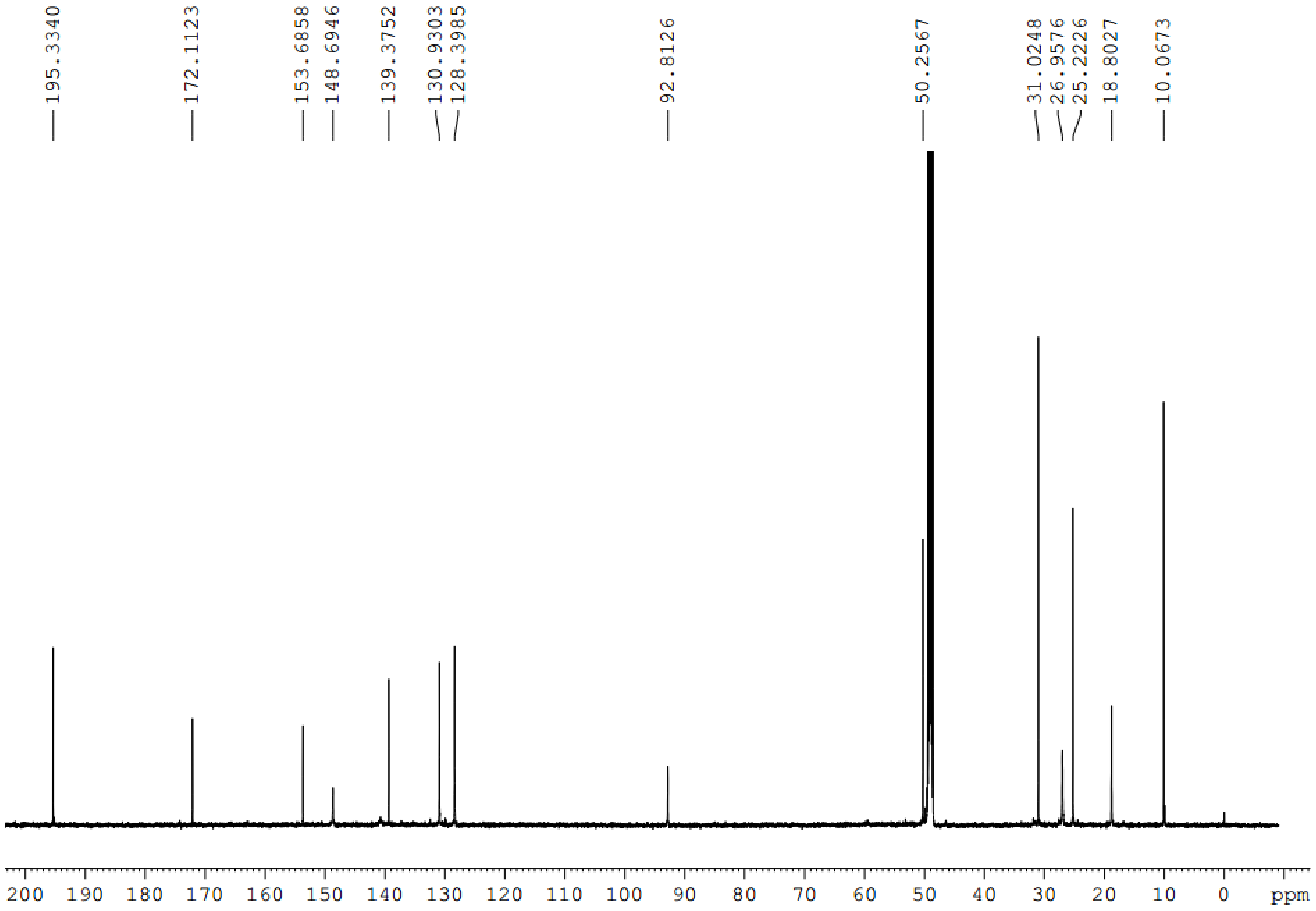

Figure S5. ^{13}C NMR (150 MHz) spectrum of compound **(+)-1** in CD_3OD

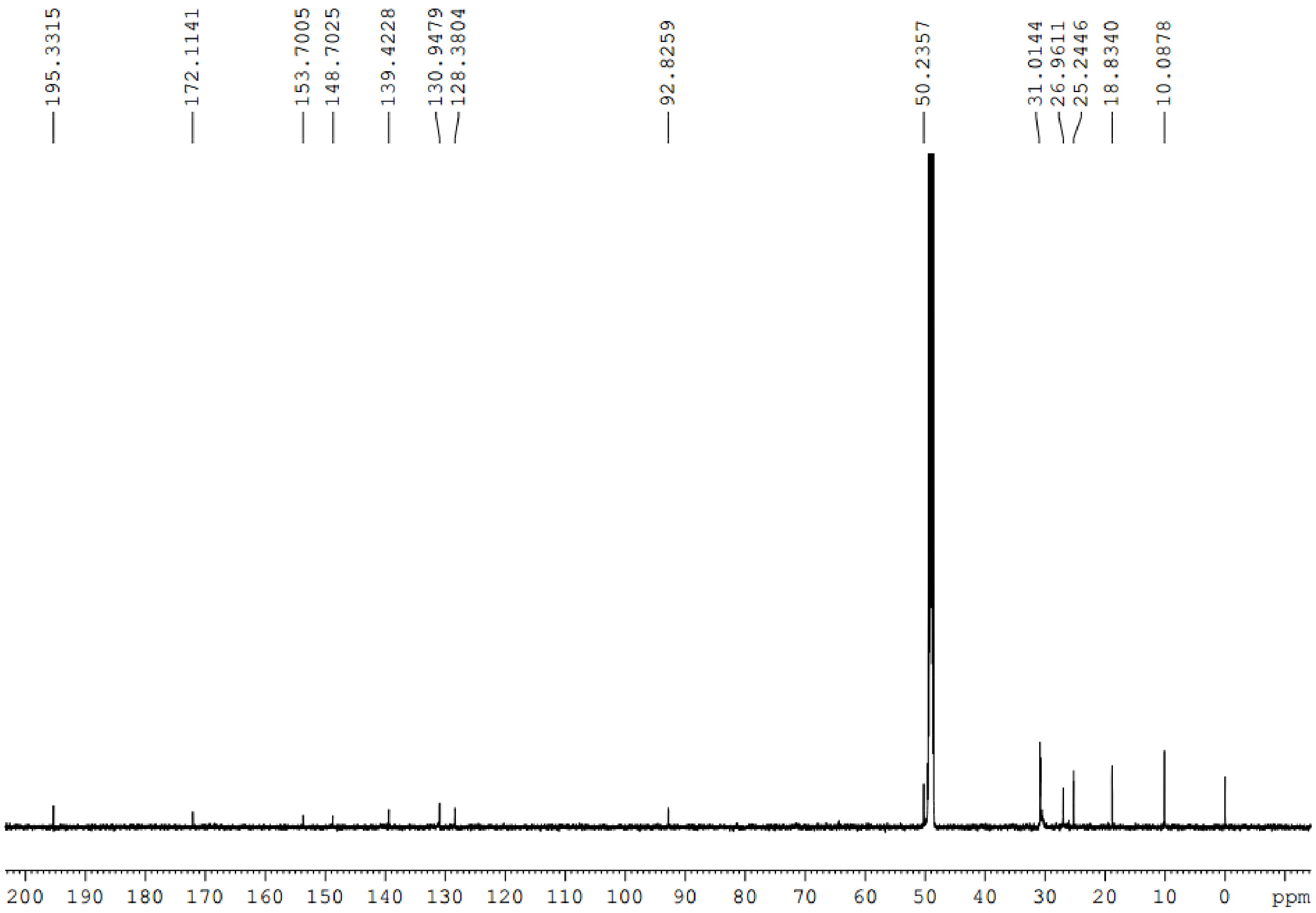

Figure S6. ^{13}C NMR (150 MHz) spectrum of compound **(-)-1** in CD_3OD

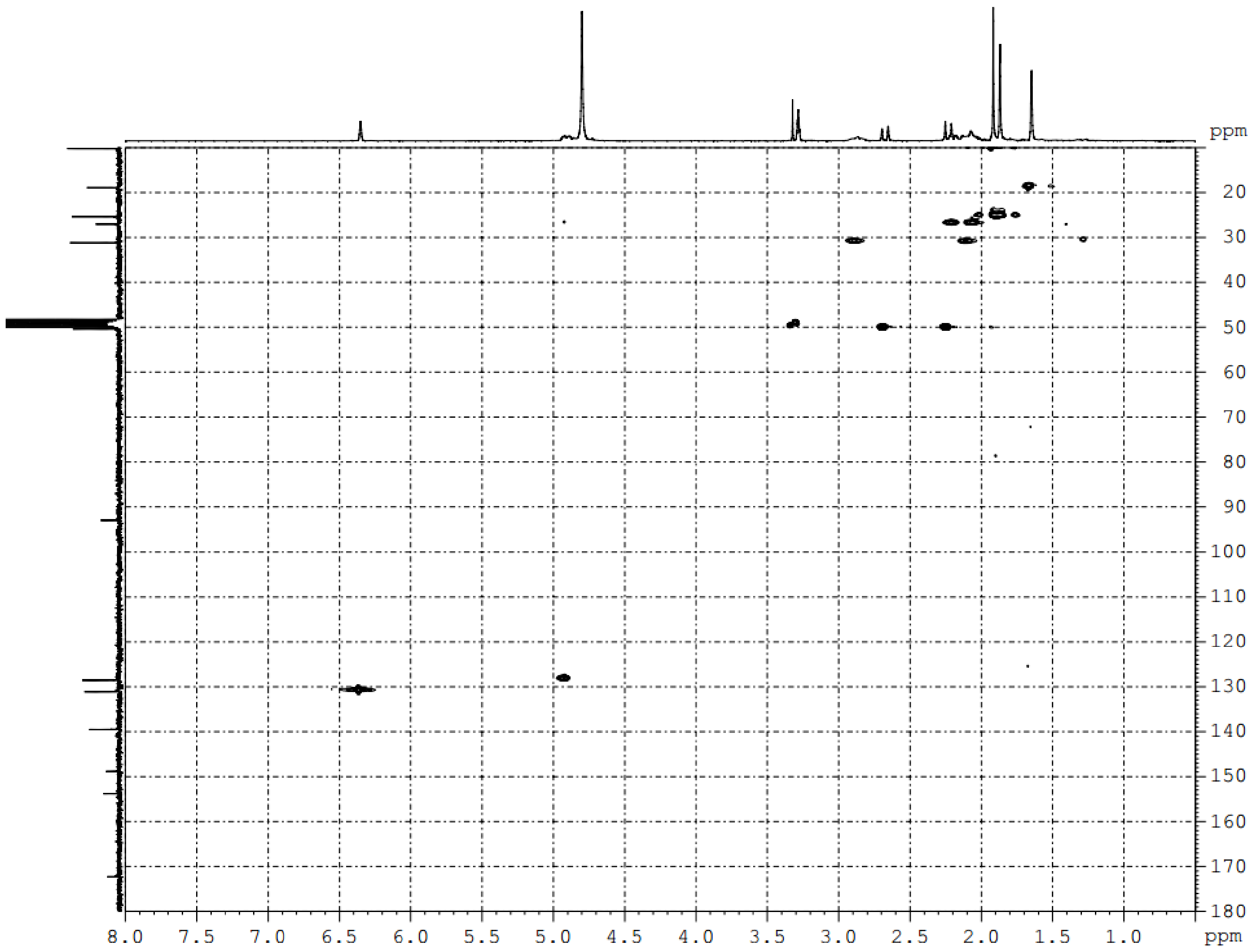

Figure S7. HSQC spectrum of compound **1** in CD_3OD

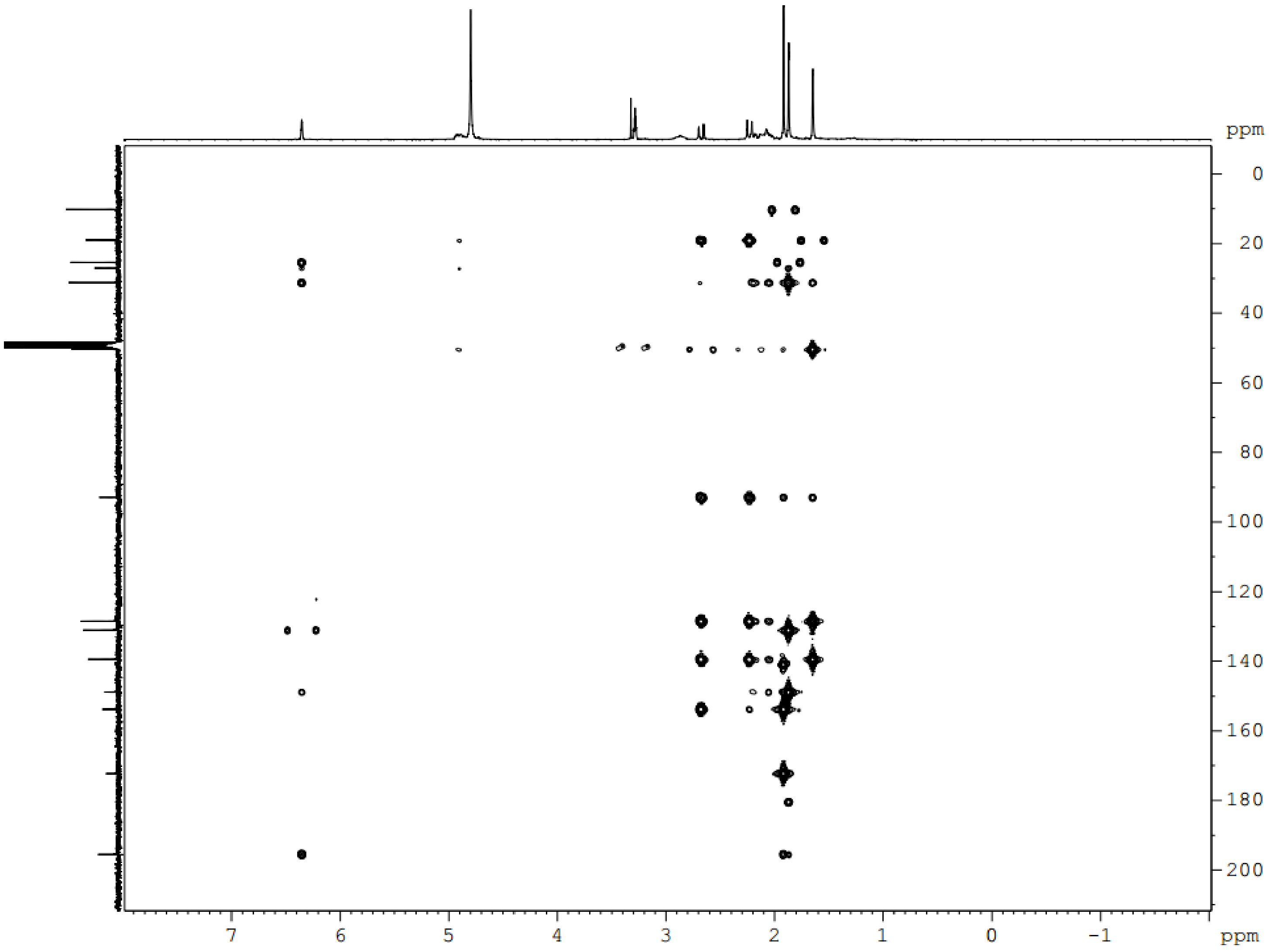

Figure S8. HMBC spectrum of compound **1** in CD_3OD

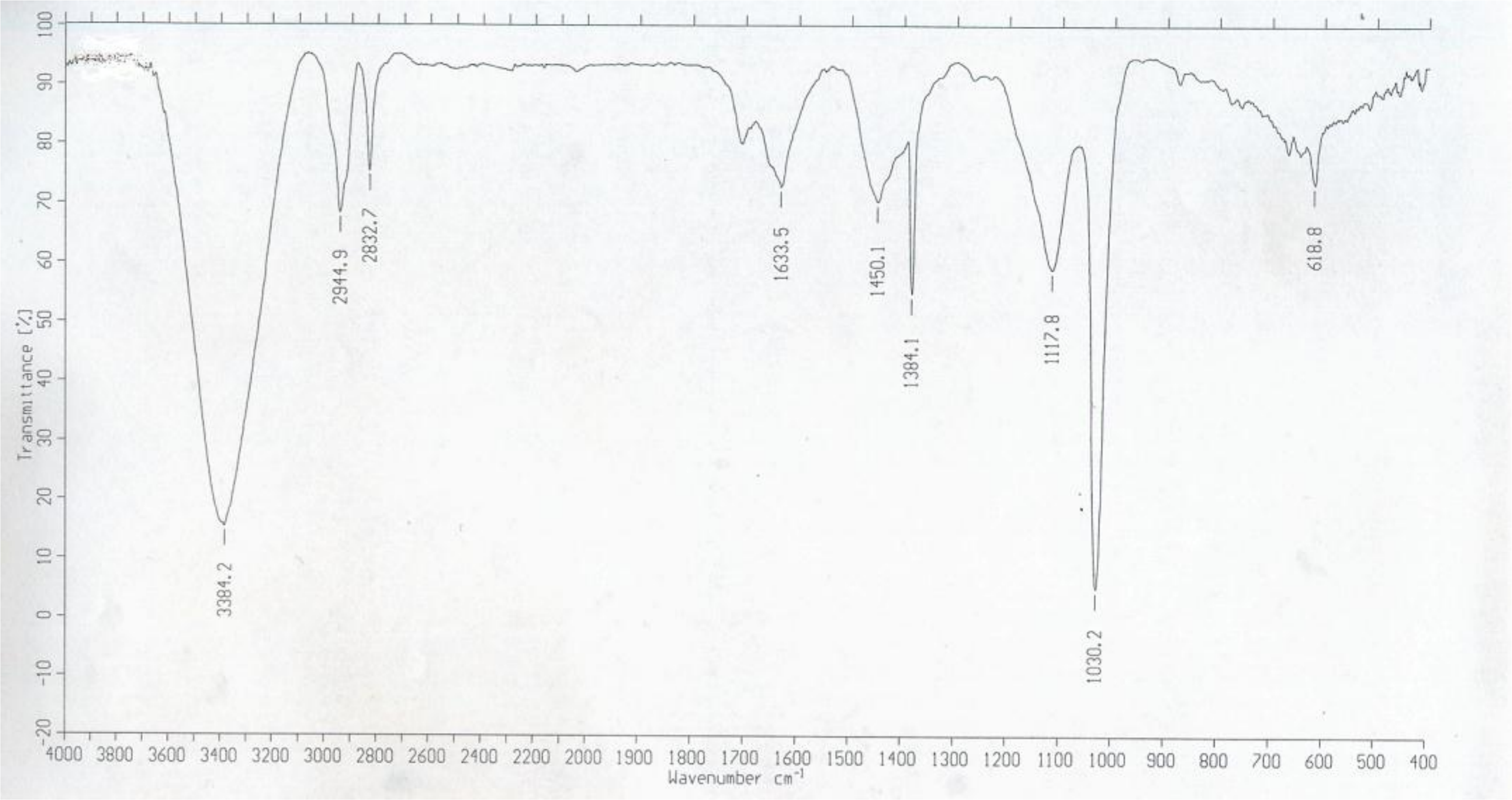

Figure S9. IR spectrum of compound **1**

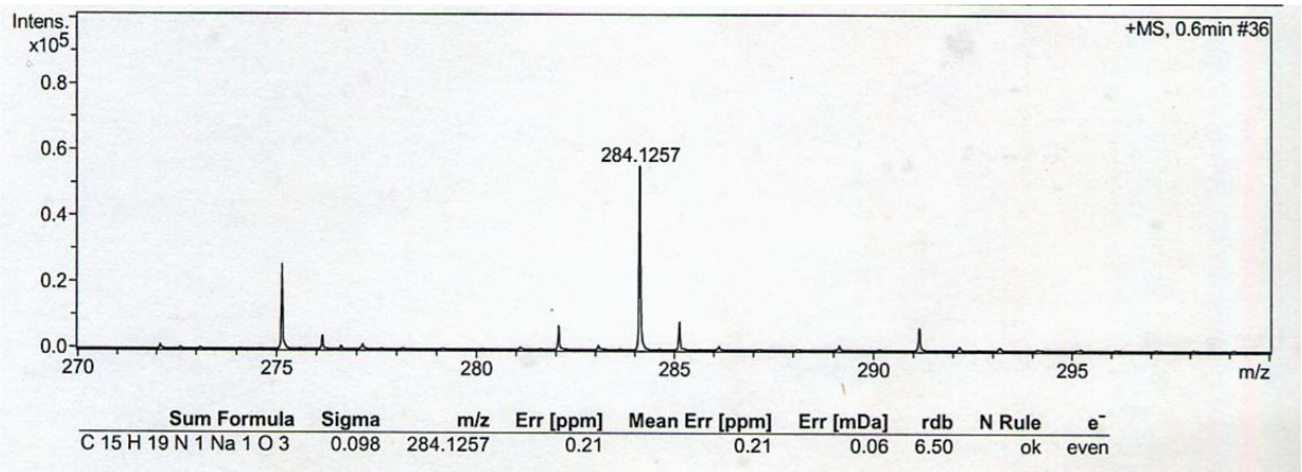

Figure S10. HRESIMS data of compound **1**

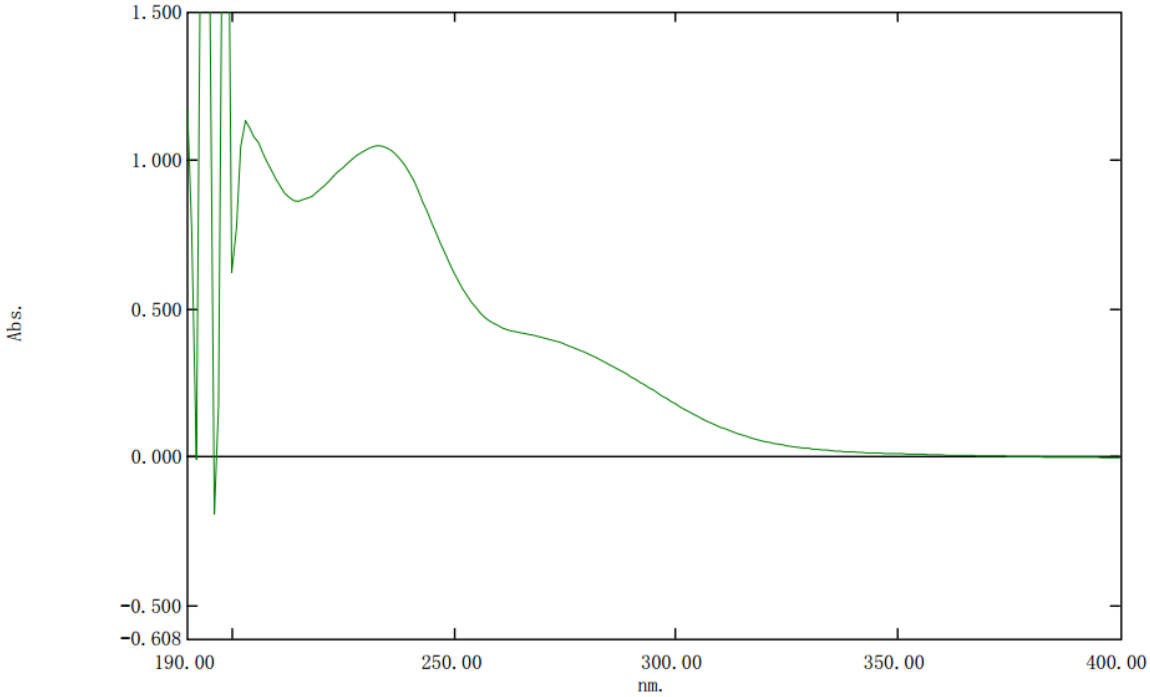

Figure S11. UV spectrum of compound **1**

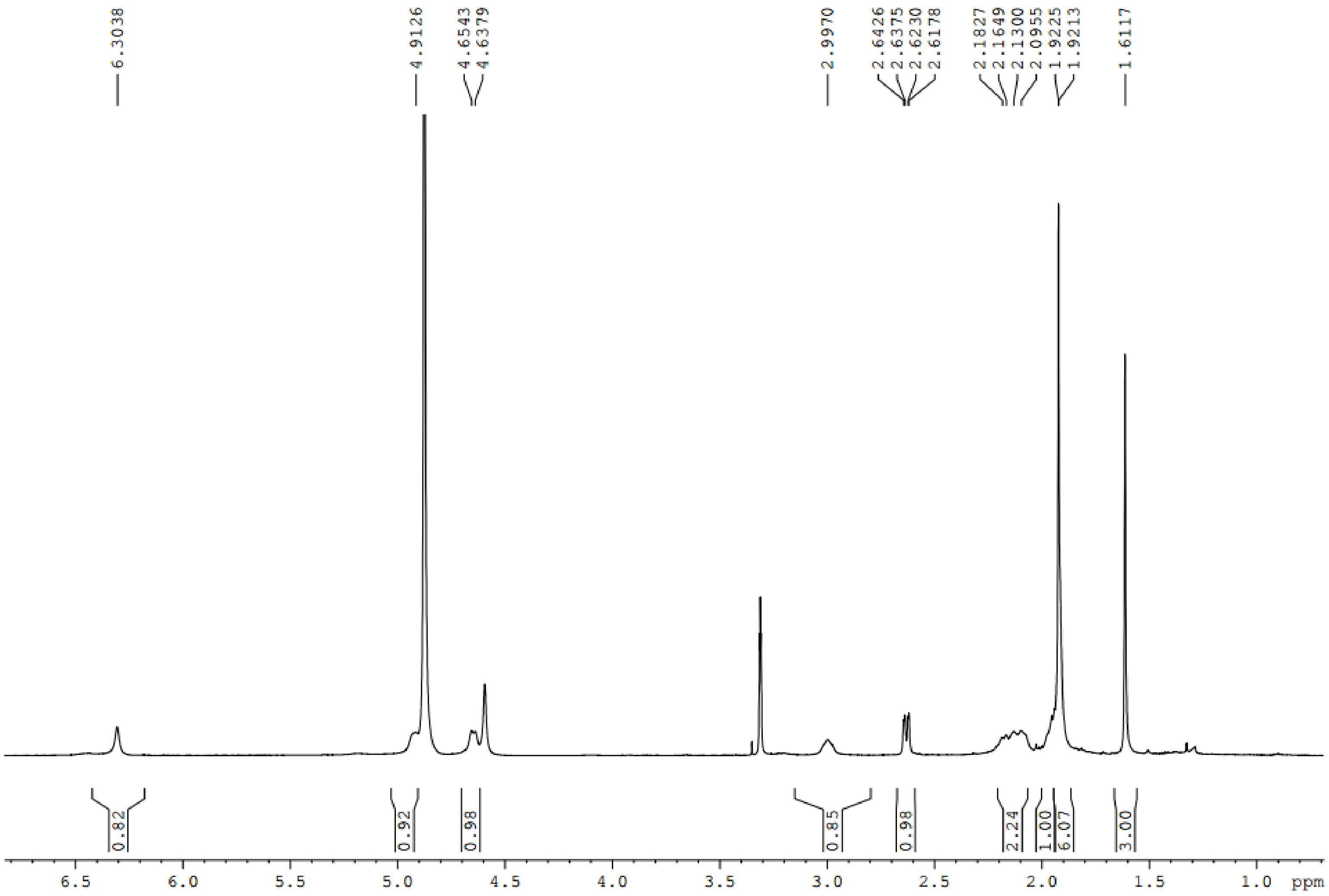

Figure S12. ^{1}H NMR (600 MHz) spectrum of compound **2** in CD_3OD

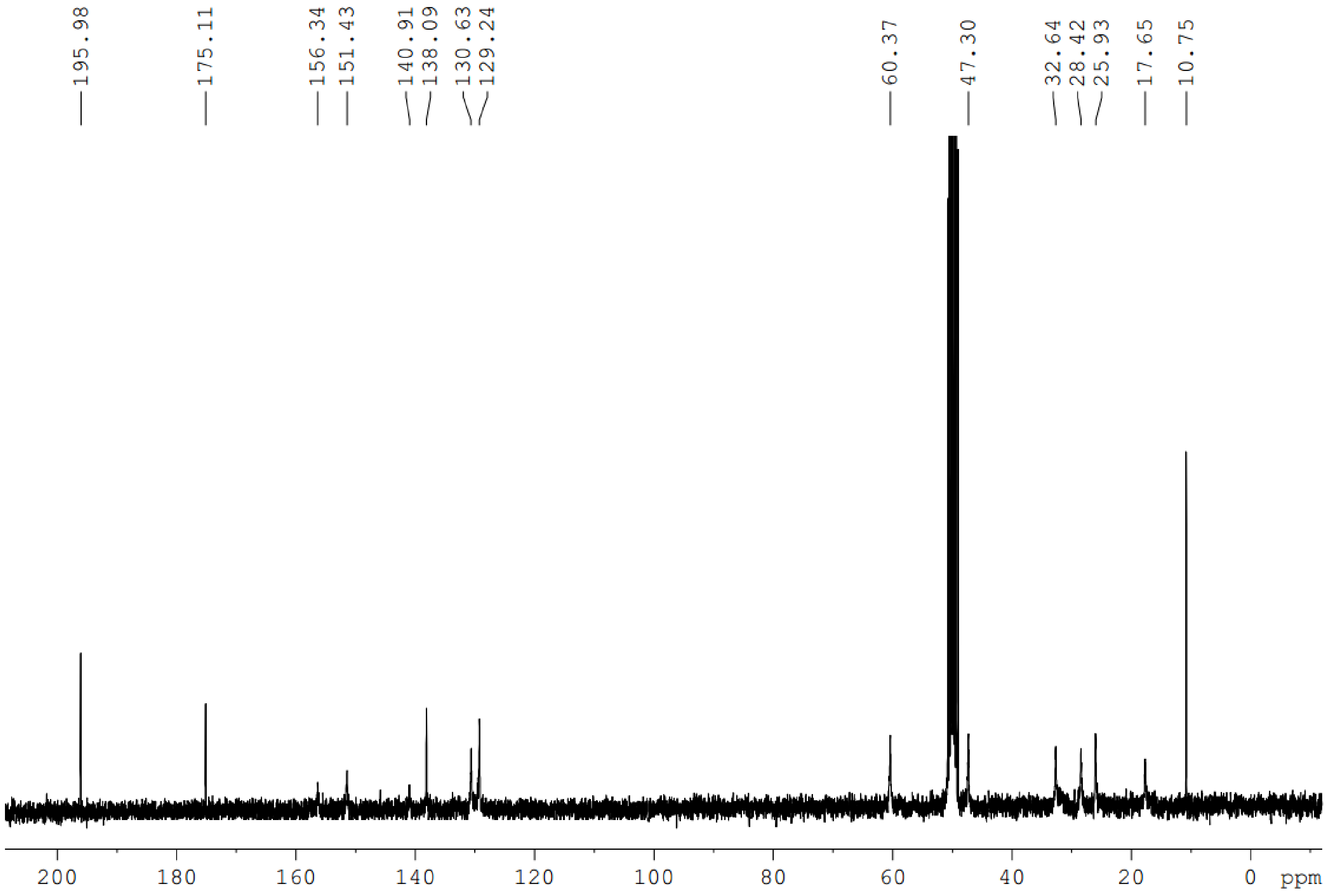

Figure S13. ^{13}C NMR (75 MHz) spectrum of compound **2** in CD_3OD

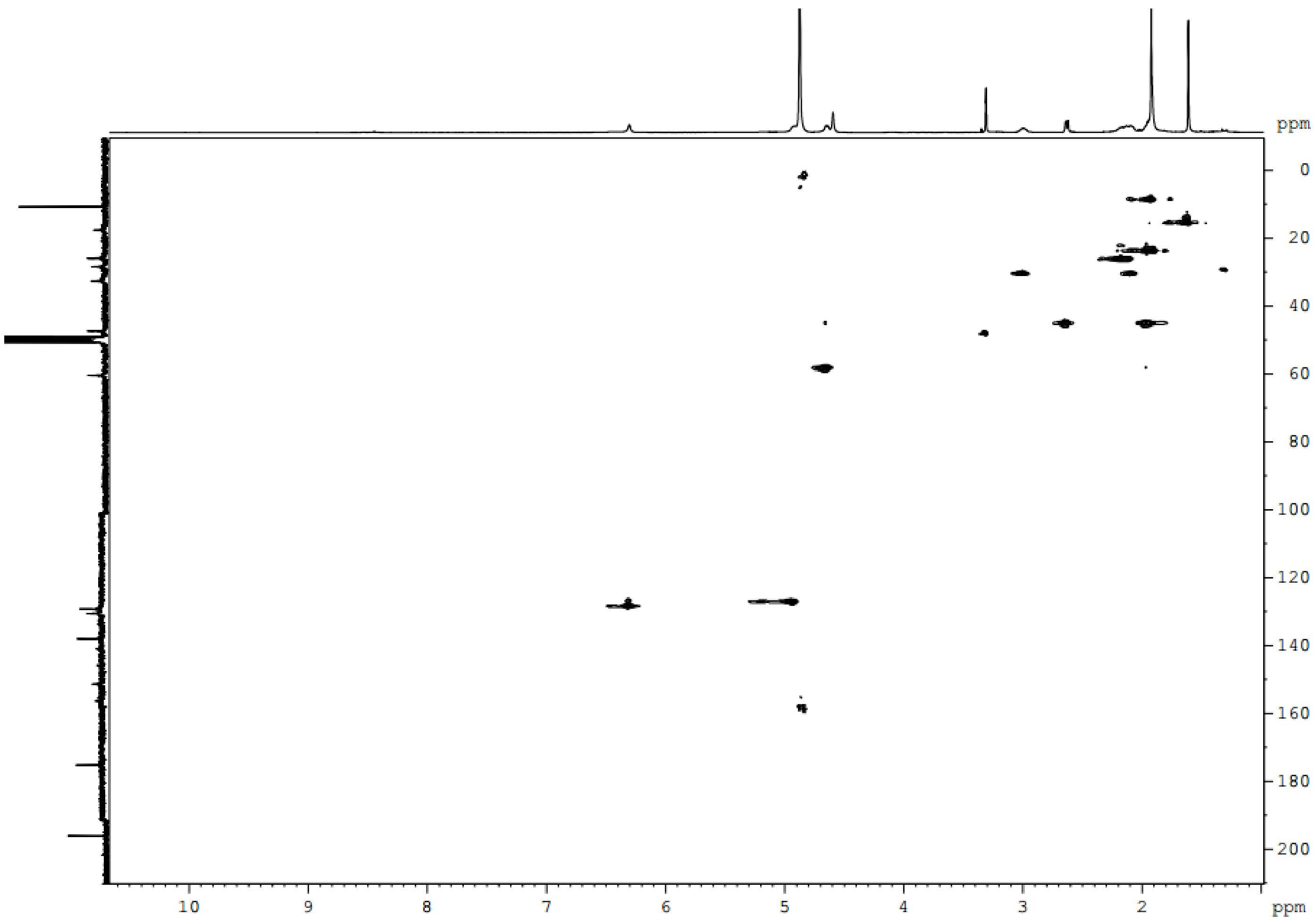

Figure S14. HSQC spectrum of compound **2** in CD_3OD

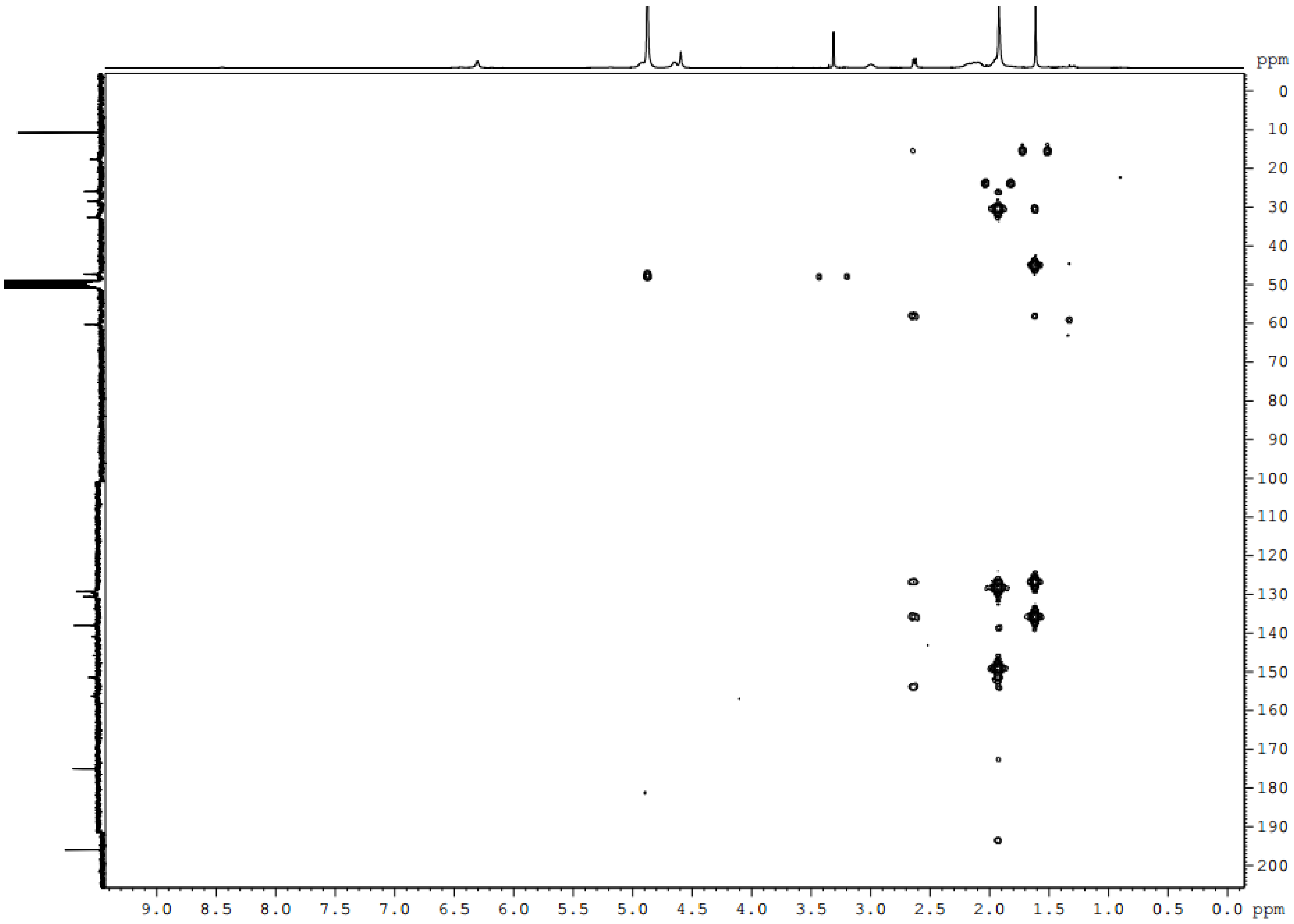

Figure S15. HMBC spectrum of compound **2** in CD_3OD

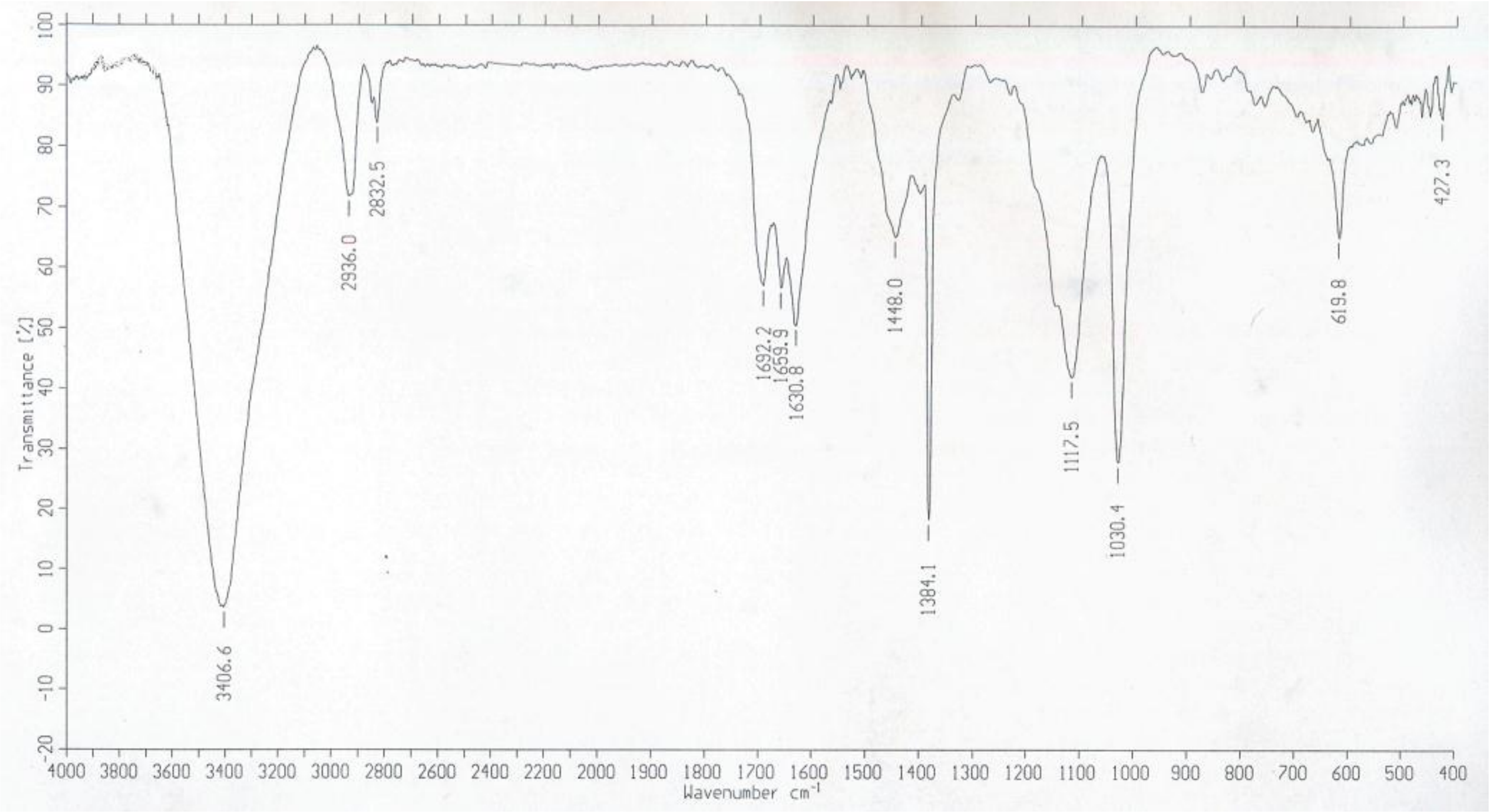

Figure S16. IR spectrum of compound **2**

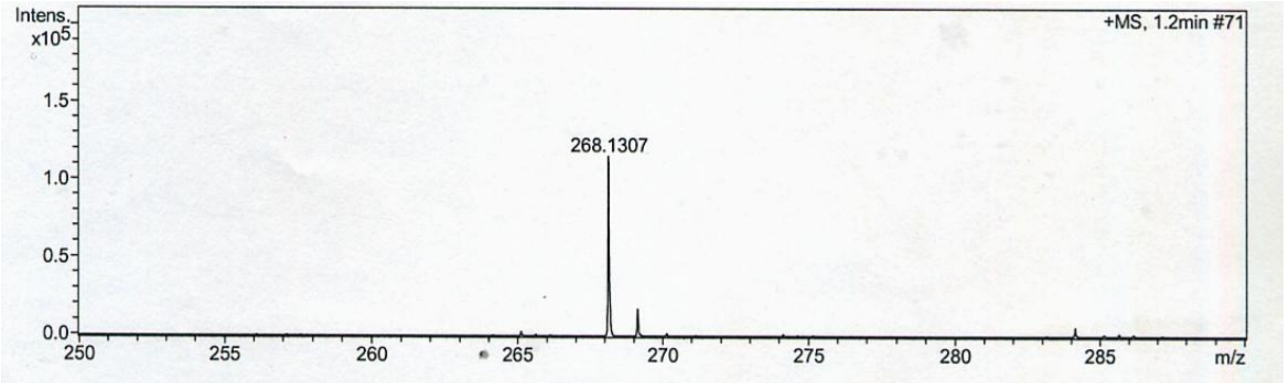

Sum Formula	Sigma	m/z	Err [ppm]	Mean Err [ppm]	Err [mDa]	rdb	N Rule	e⁻
C 15 H 19 N 1 Na 1 O 2	0.098	268.1308	0.28	0.28	0.07	6.50	ok	even

Figure S17. HRESIMS data of compound **2**

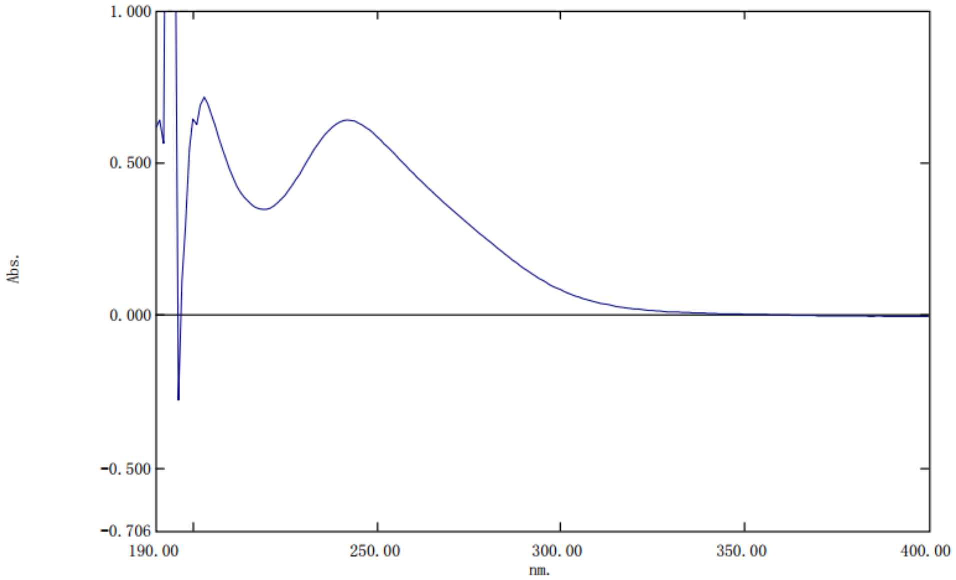

Figure S18. UV spectrum of compound **2**

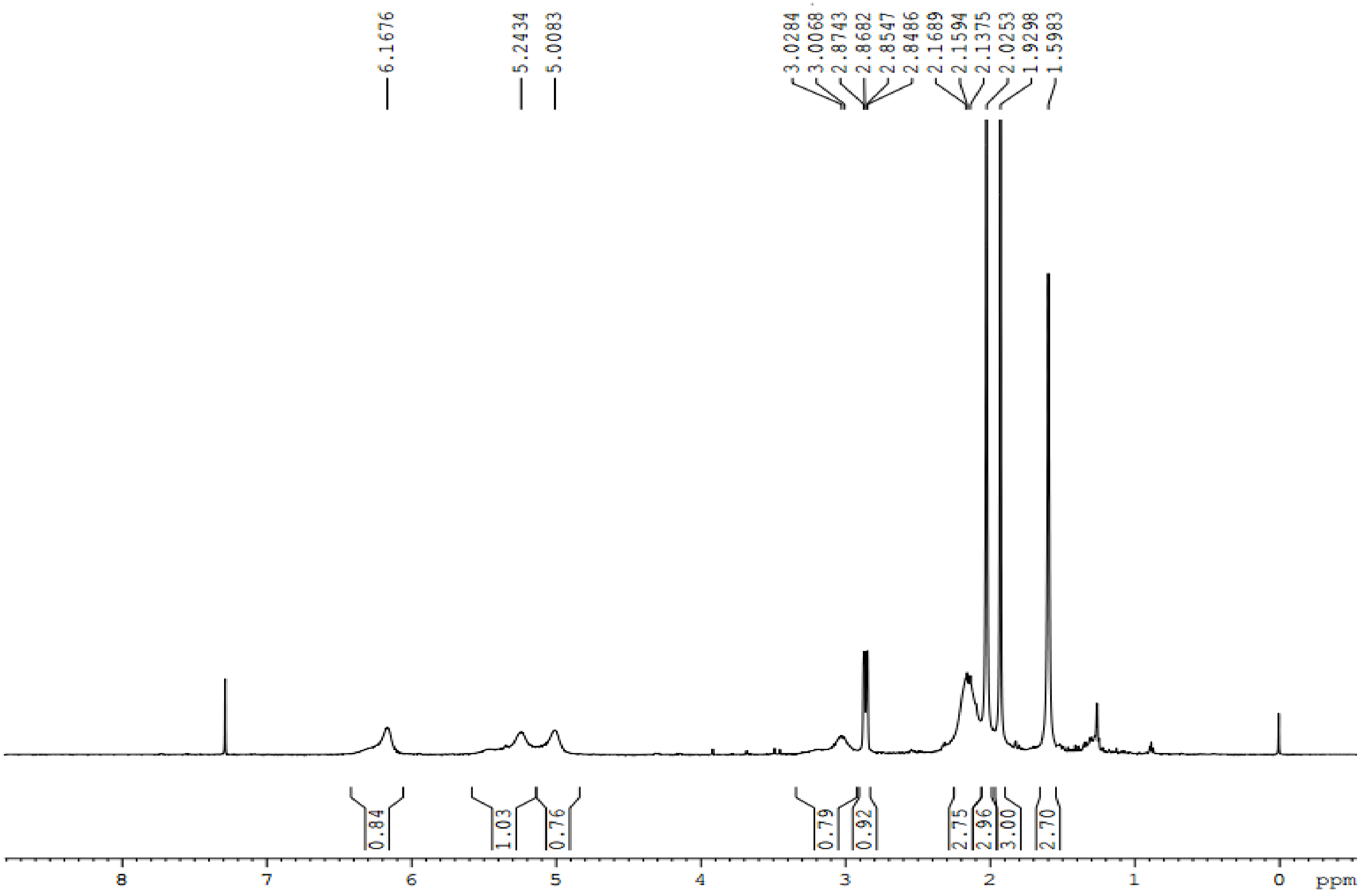

Figure S19. ^{1}H NMR (600 MHz) spectrum of compound **3** in $CDCl_3$

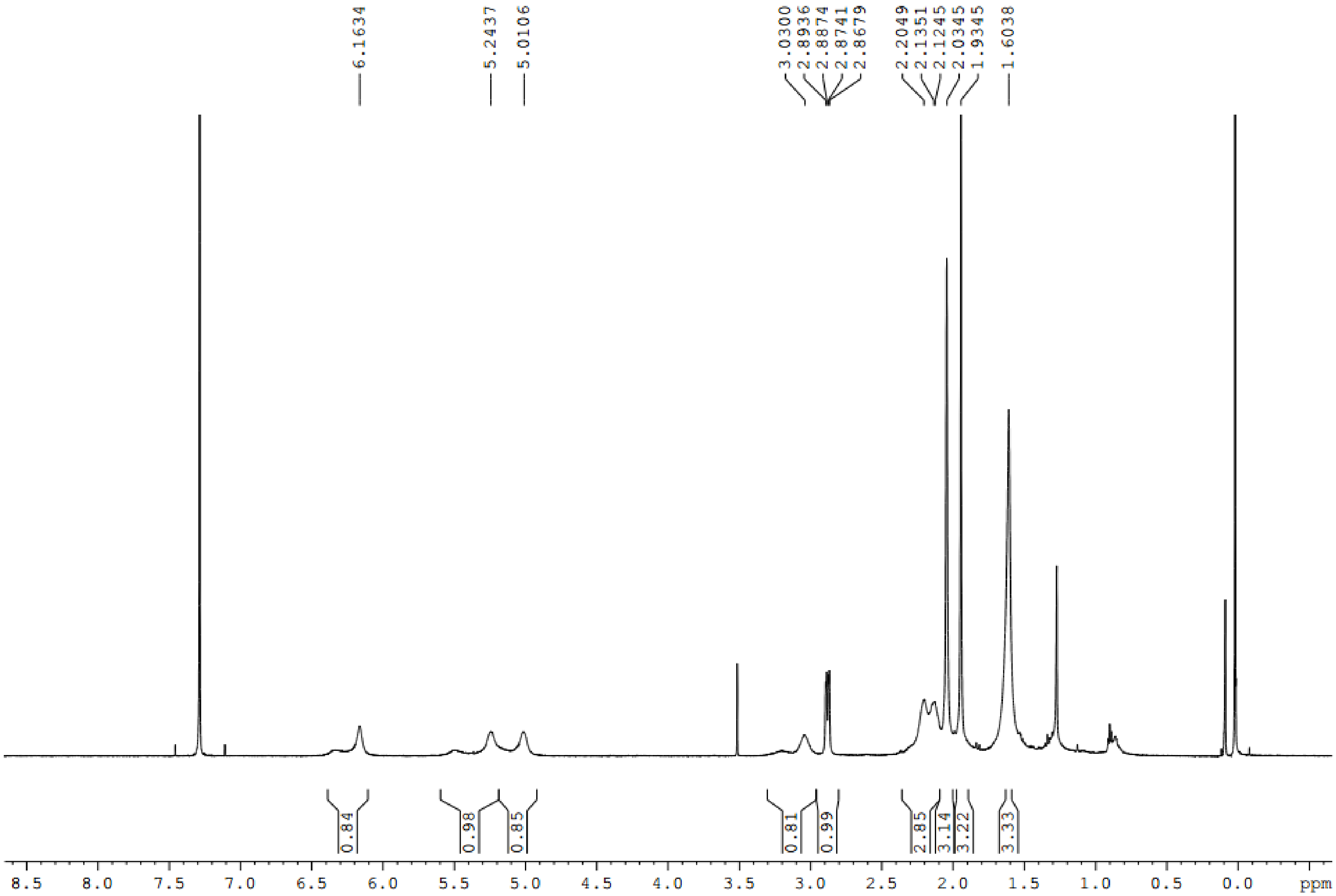

Figure S20. ^{1}H NMR (600 MHz) spectrum of compound **(+)-3** in $CDCl_3$

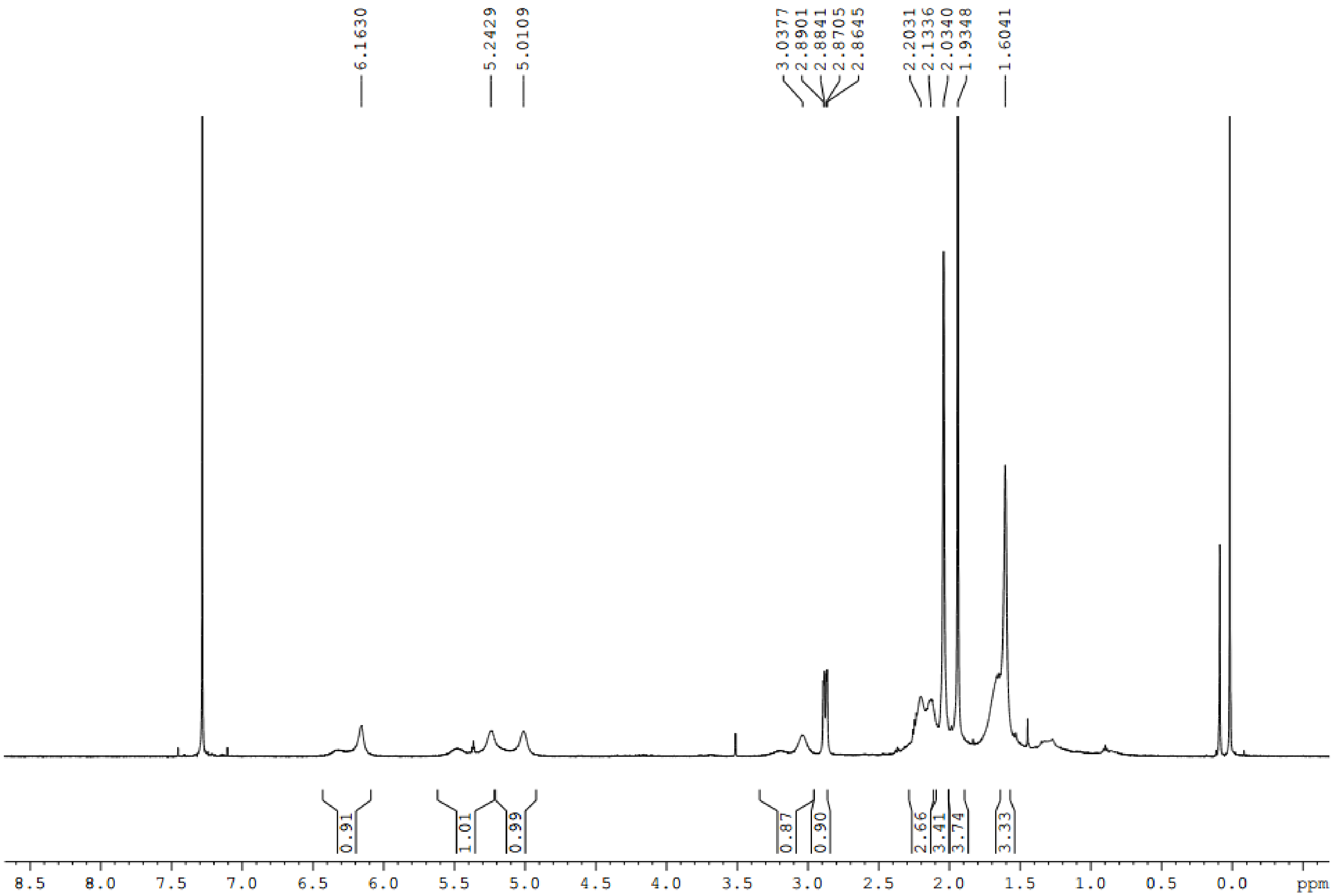

Figure S21. ^{1}H NMR (600 MHz) spectrum of compound **(-)-3** in $CDCl_3$

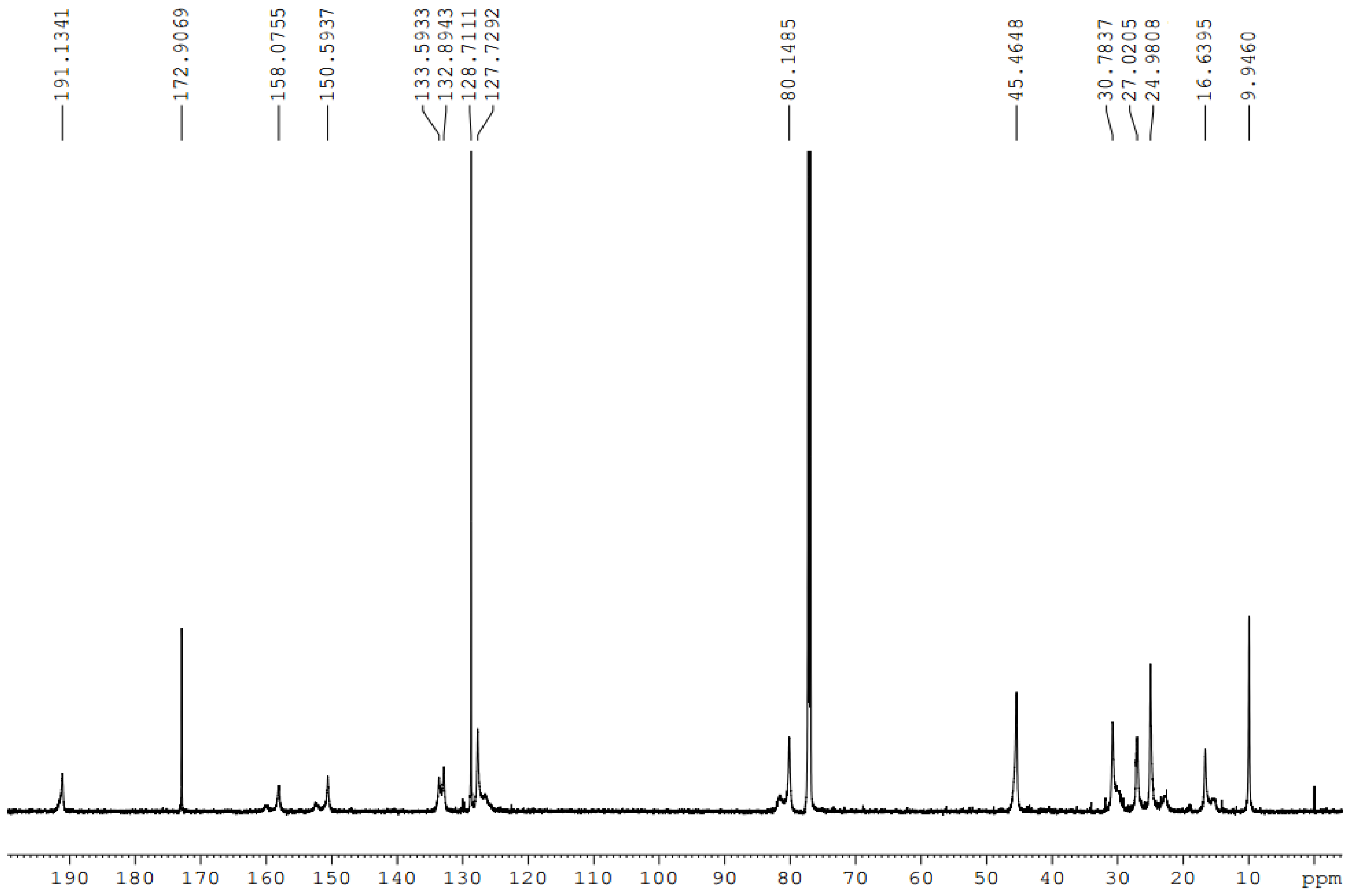

Figure S22. ^{13}C NMR (150 MHz) spectrum of compound **3** in $CDCl_3$

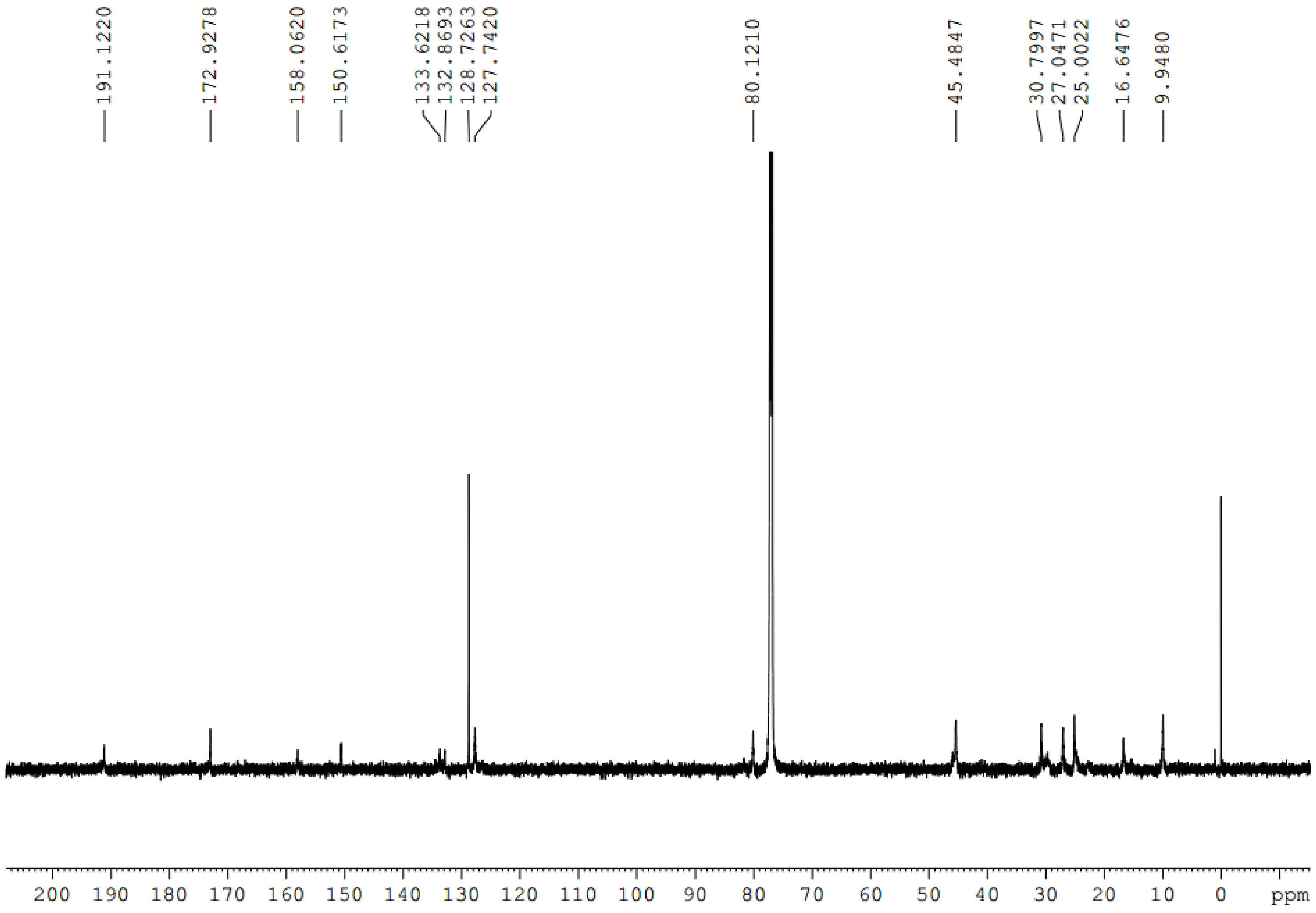

Figure S23. ^{13}C NMR (150 MHz) spectrum of compound **(+)-3** in $CDCl_3$

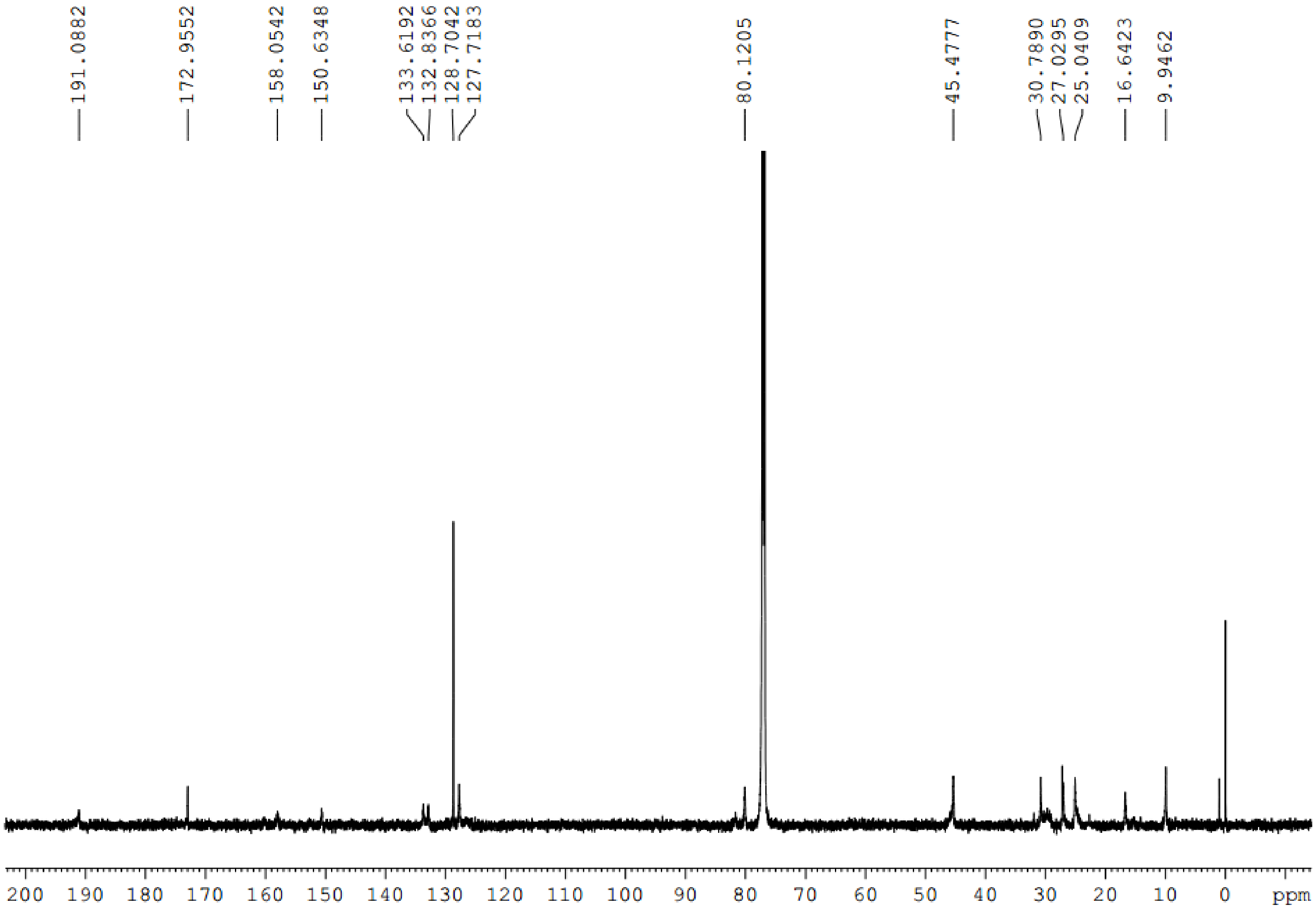

Figure S24. ^{13}C NMR (150 MHz) spectrum of compound **(-)-3** in $CDCl_3$

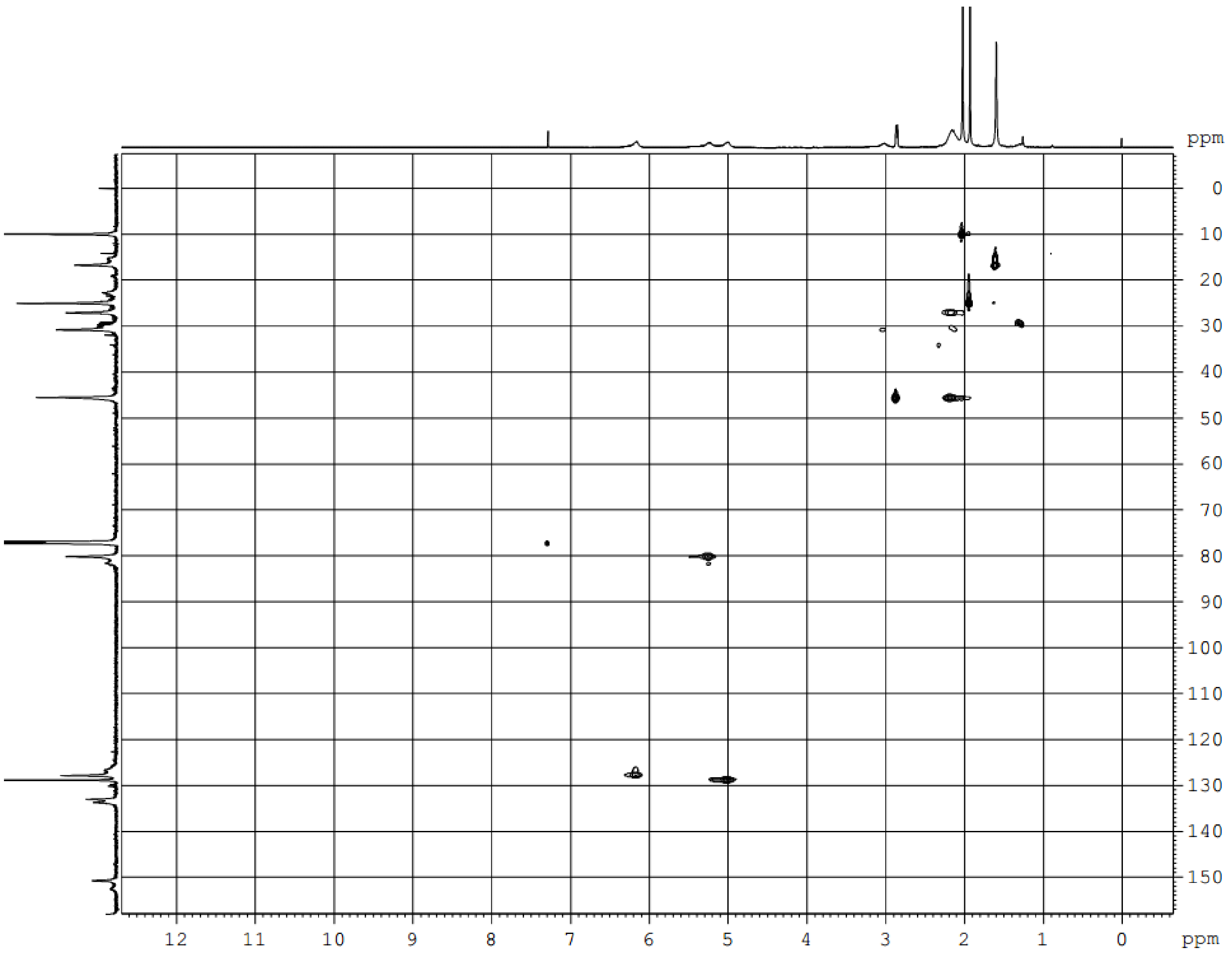

Figure S25. HSQC spectrum of compound **3** in $CDCl_3$

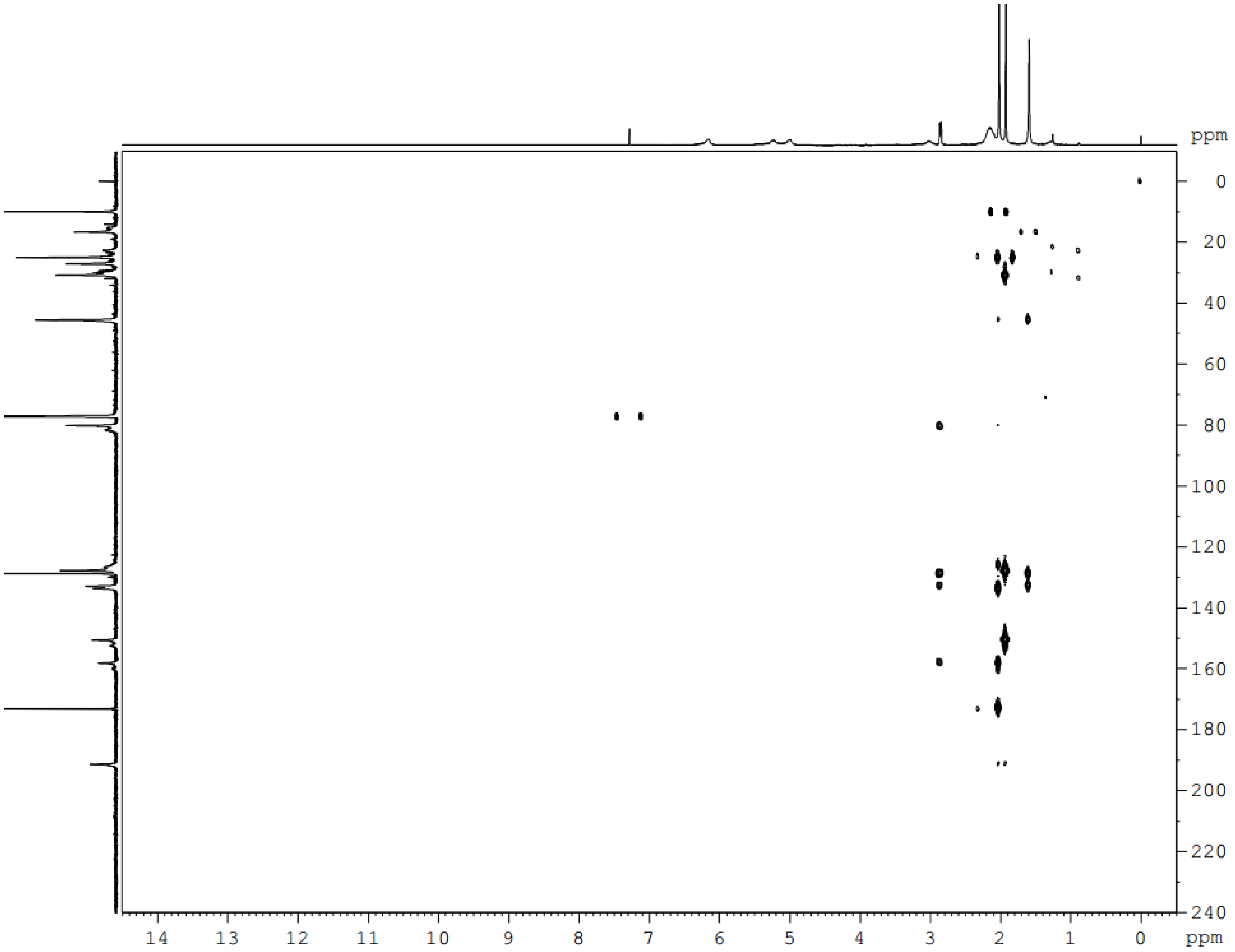

Figure S26. HMBC spectrum of compound **3** in $CDCl_3$

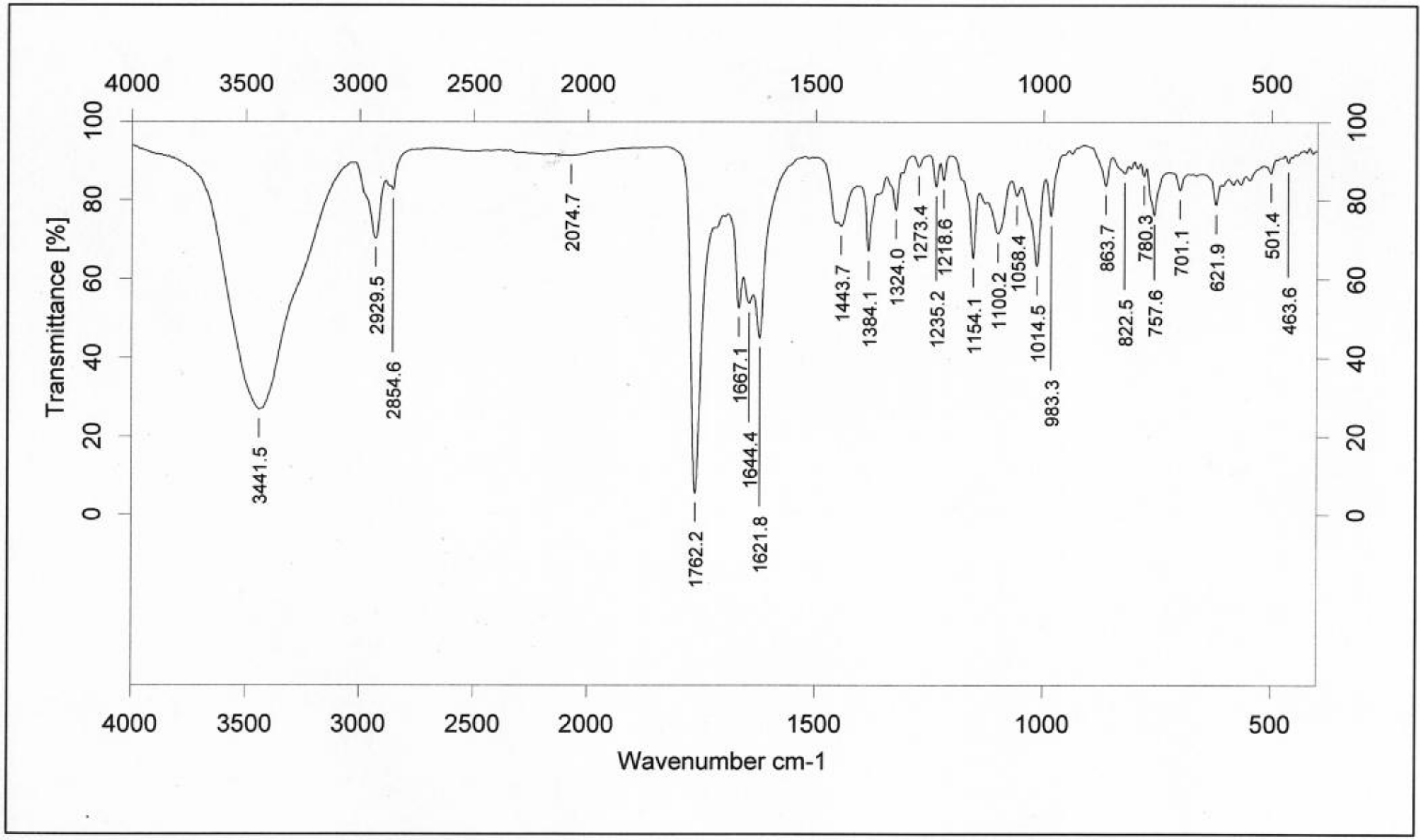

Figure S27. IR spectrum of compound **3**

Mass	Calc. Mass	mDa	PPM	DBE	Formula	i-FIT	i-FIT Norm	Fit Conf %	C	H	O
247.1336	247.1334	0.2	0.8	6.5	C15 H19 O3	57.7	n/a	n/a	15	19	3

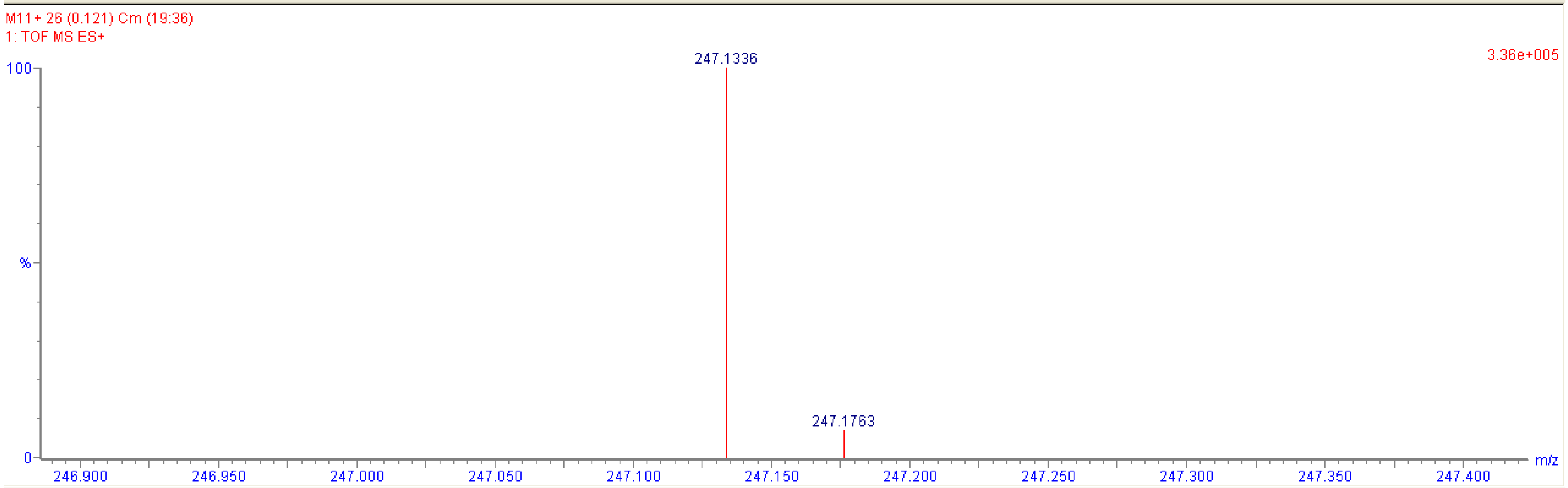

Figure S28. HRESIMS data of compound **3**

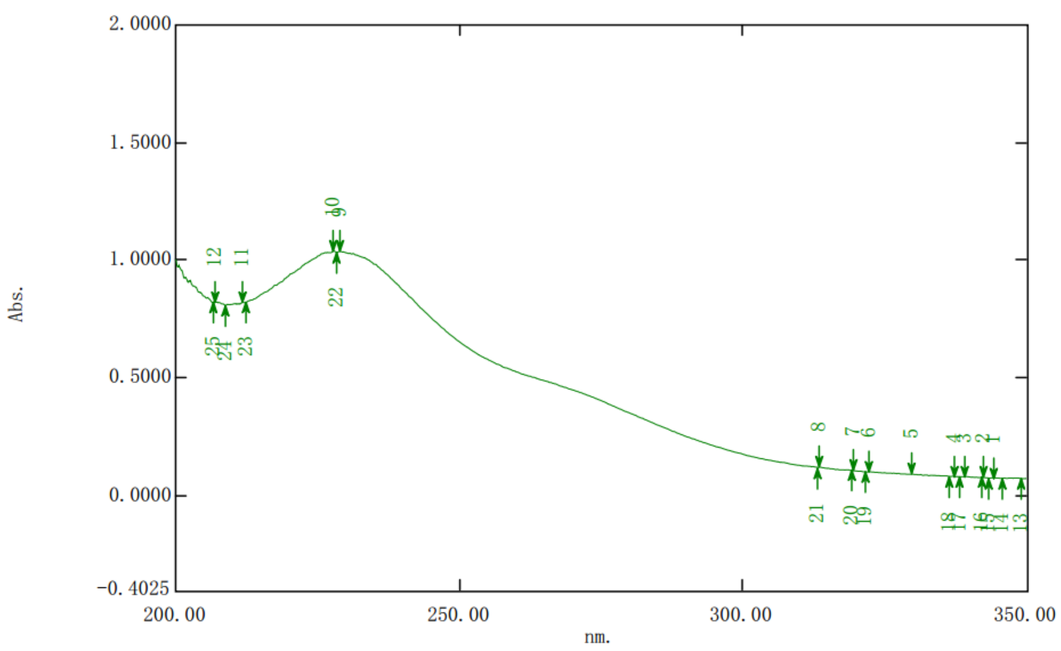

Figure S29. UV spectrum of compound **3**

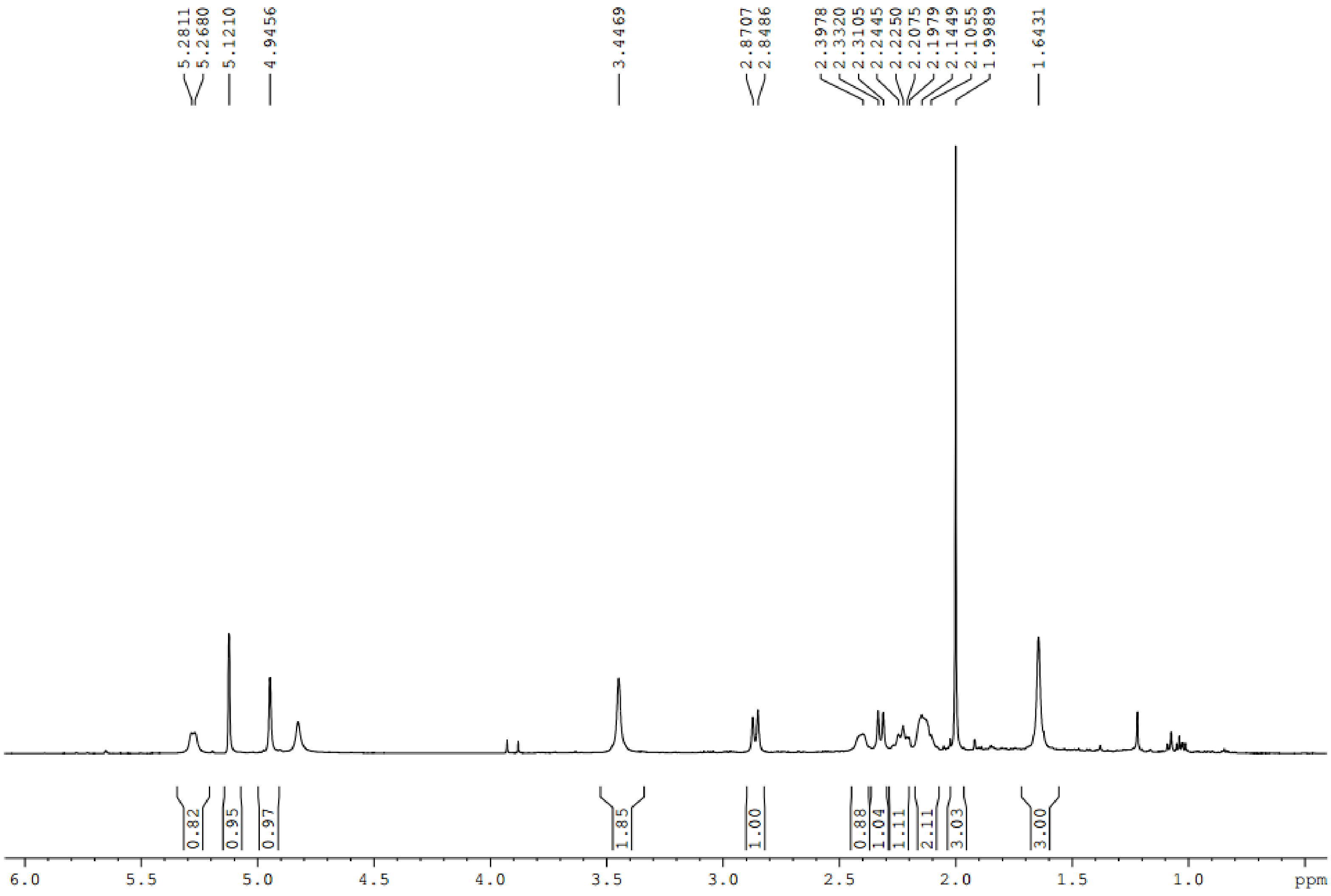

Figure S30. ^{1}H NMR (600 MHz) spectrum of compound **4** in $CDCl_3$

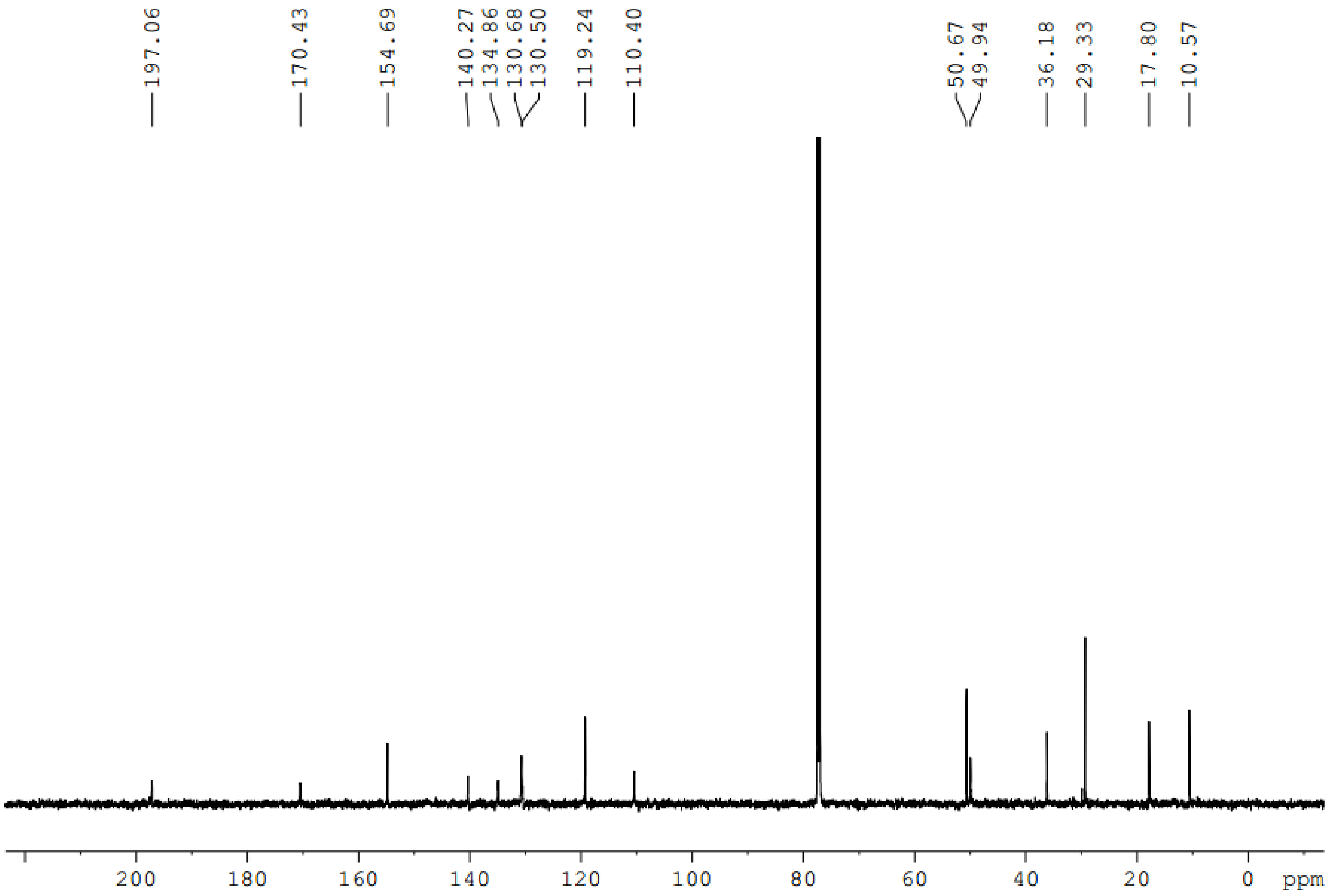

Figure S31. ^{13}C NMR (150 MHz) spectrum of compound **4** in $CDCl_3$

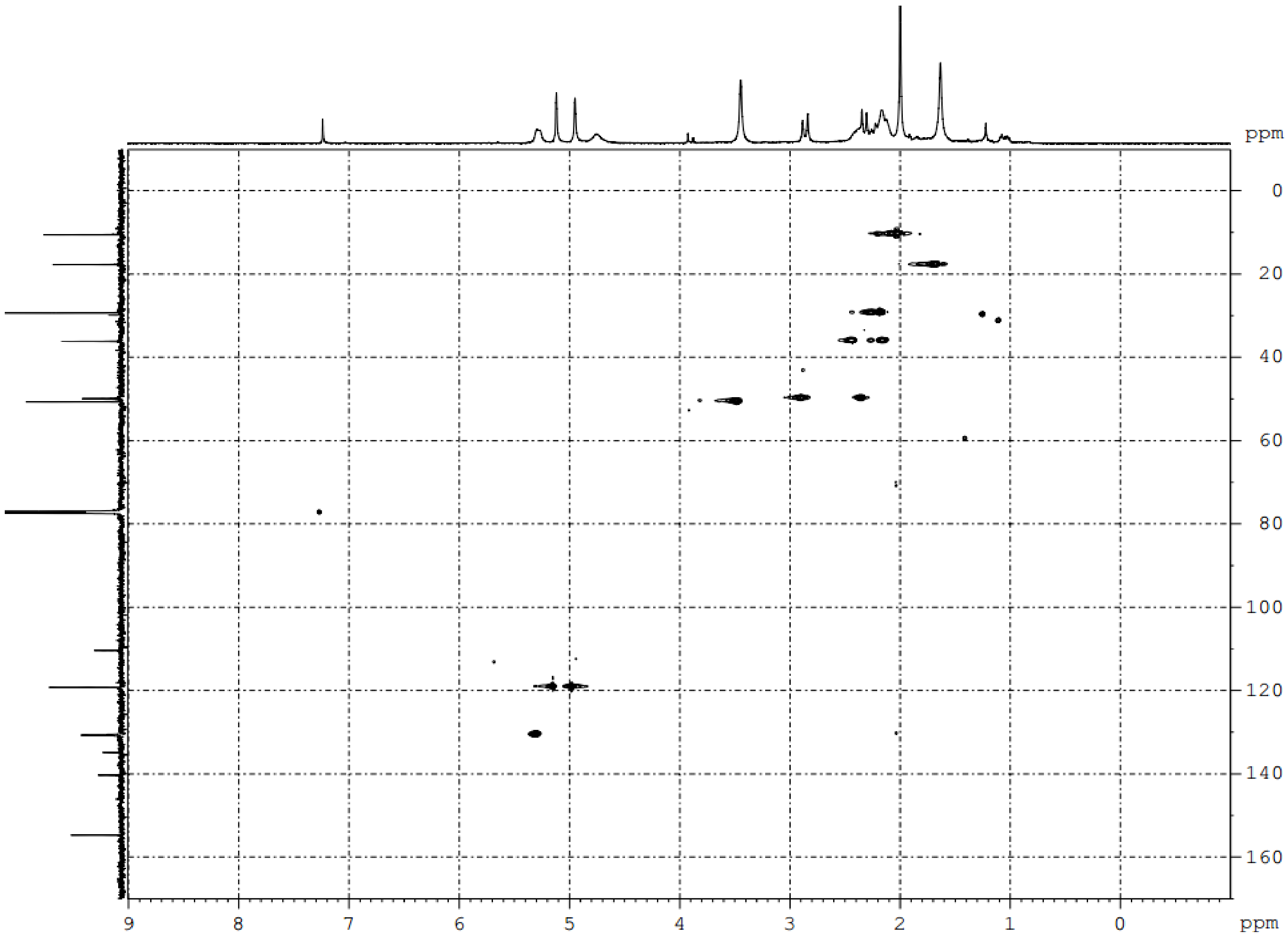

Figure S32. HSQC spectrum of compound **4** in $CDCl_3$

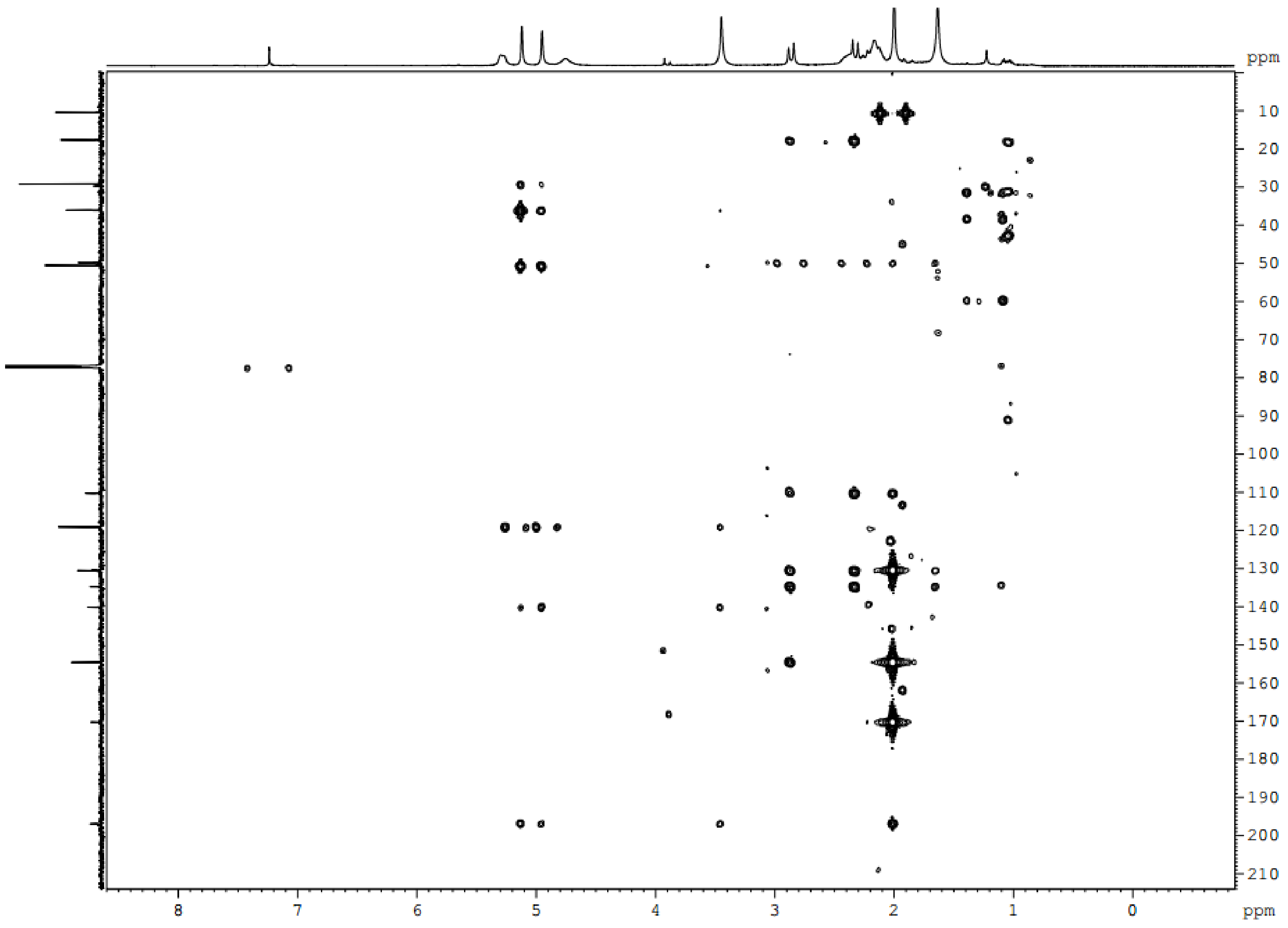

Figure S33. HMBC spectrum of compound **4** in $CDCl_3$

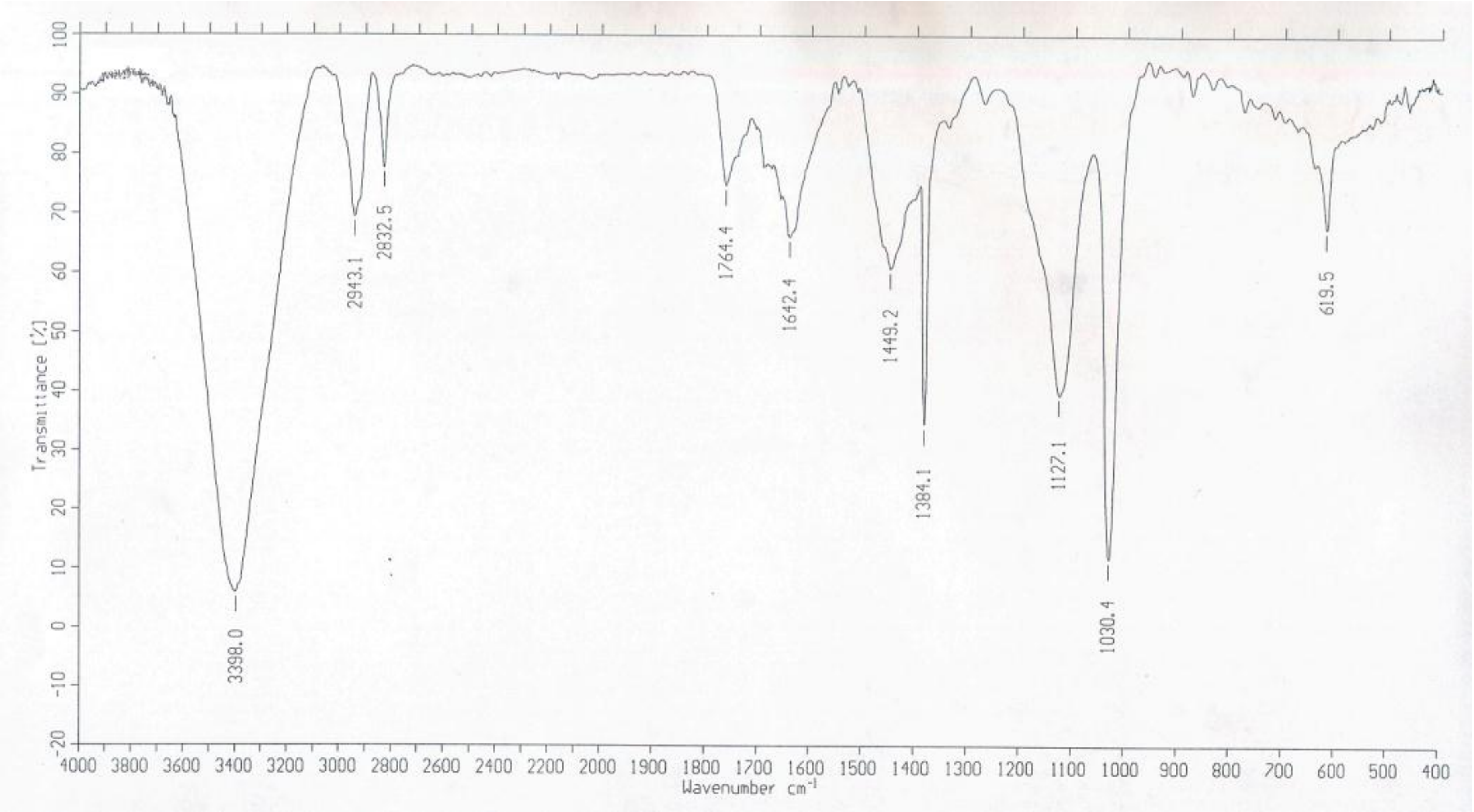

Figure S34. IR spectrum of compound **4**

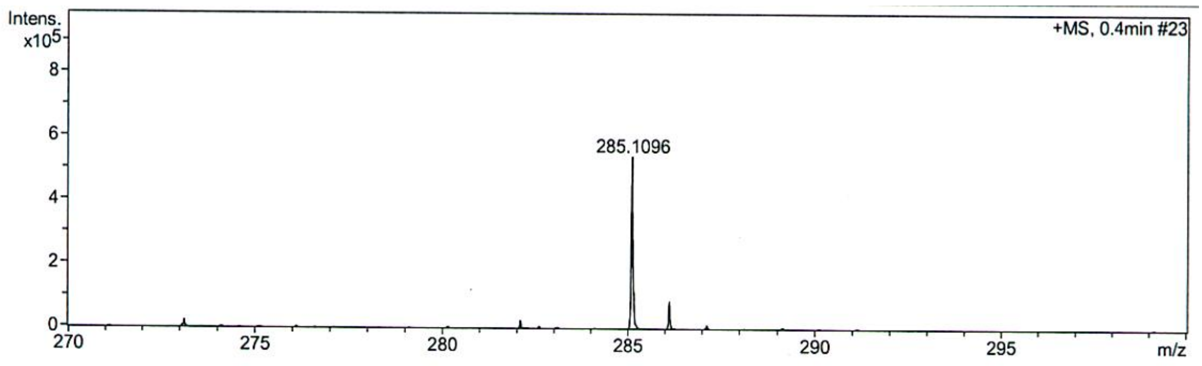

Sum Formula	Sigma	m/z	Err [ppm]	Mean Err [ppm]	Err [mDa]	rdb	N Rule	e⁻
C 15 H 18 Na 1 O 4	0.097	285.1097	0.63	0.63	0.18	6.50	ok	even

Figure S35. HRESIMS data of compound **4**

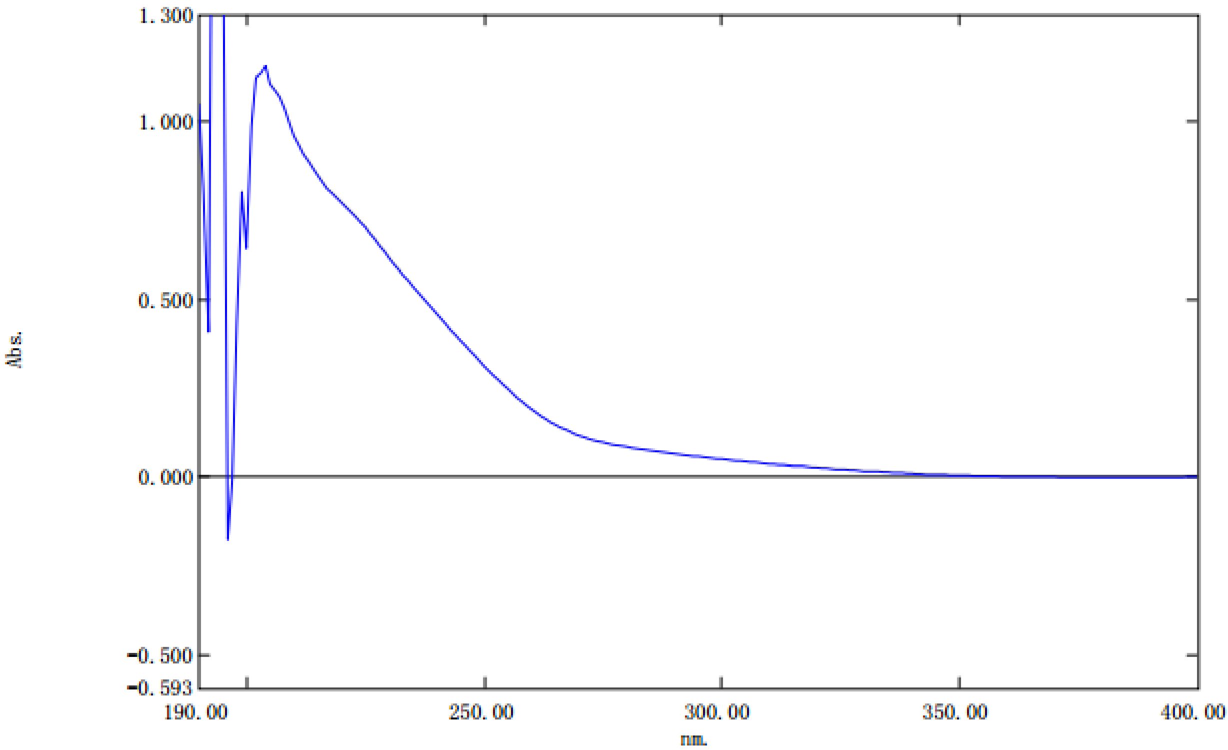

Figure S36. UV spectrum of compound **4**

Single-Crystal X-ray Diffraction Analysis and Crystallographic Data of Compounds 1, (-)-1, 2, 3, (+)-3, (-)-3, and 5.

Diffraction intensity data for compounds **1**, **(-)-1**, **2**, **3**, and **5** were acquired on a Sapphire CCD with a graphite monochromated Cu Kα radiation, λ = 1.54184 Å at 173.01 (10) K. The structures were solved by direct methods using the SHELXS-97 program, and refined by the program SHELXL-97 and full-matrix least-squares calculations. Data for compounds **(+)-3** and **(-)-3** were acquired on an Xcalibur, Eos, Gemini diffractometer using monochromatized Cu Kα radiation, λ = 1.54184 Å at 104.6 K, and the structure was solved by direct methods using SHELXL. In the structure refinements, non-hydrogen atoms were placed on the geometrically ideal positions by the "ride on" method. Hydrogen atoms bonded to oxygen were located by the structure factors with isotropic temperature factors. All of the data can be obtained free of charge from the CCDC via http://www.ccdc.cam.ac.uk/Community/Requestastructure/Pages/DataRequest.aspx.

Crystal data for **1**: colorless needles, $C_{15}H_{19}NO_3$, M = 261.31, space group orthorhombic, P b c a; unit cell dimensions were determined to be a = 12.5667 (3) Å, b = 10.7520 (3) Å, c = 19.7597 (5) Å, α = 90.00 °, β = 90.00 °, γ = 90.00 °, V = 2669.86 (11) Å^3, Z = 8, Dx = 1.300 mg/m^3, F (000) = 1120, μ (Cu Kα) = 0.733 mm^{-1}. The final indices R_1 = 0.0401, wR_2 = 0.1130. Reflections collected: 6701. (CCDC No. 1486405).

Crystal data for (-)-**1**: colorless needles, $C_{15}H_{19}NO_3$, 0.17 (H_2O), M = 264.31, space group monoclinic, P 3_1 2 1; unit cell dimensions were determined to be a = 9.64797 (11) Å, b = 9.64797 (11) Å, c = 26.9006 (3) Å, α = 90.00 °, β = 90.00 °, γ = 120.00 °, V = 2168.53 (4) Å^3, Z = 6, Dx = 1.214 mg/m^3, F (000) = 850, μ (Cu Kα) = 0.691 mm^{-1}. The final indices R_1 = 0.0287, wR_2 = 0.0771. Reflections collected: 23169. Flack parameter = 0.1 (2). (CCDC No. 1486406).

Crystal data for **2**: colorless needles, $C_{15}H_{19}NO_2$, M = 245.31, space group monoclinic, C 1 2/c 1; unit cell dimensions were determined to be a = 17.9393 (4) Å, b = 10.02516 (19) Å, c = 14.8144 (3) Å, α = 90.00 °, β = 104.106 (2) °, γ = 90.00 °, V = 2583.96 (9) Å^3, Z = 8, Dx = 1.261 mg/m^3, F (000) = 1056, μ (Cu Kα) = 0.663 mm^{-1}. The final indices R_1 = 0.0359, wR_2 = 0.0956. Reflections collected: 20030. (CCDC No. 1486407).

Crystal data for **3**: colorless cube crystals, $C_{15}H_{18}O_3$; M = 246.29, space group orthorhombic, P b c a; unit cell dimensions were determined to be a = 12.13063 (16) Å, b = 10.56530 (15) Å, c = 19.8399 (3) Å, α = 90.00 °, β = 90.00 °, γ = 90.00 °, V = 2542.75 (6) Å^3, Z = 8, Dx = 1.287 mg/m^3, F (000) = 1056, μ (Cu Kα) = 0.715 mm^{-1}. The final indices R_1 = 0.0352, wR_2 = 0.0928. Reflections collected: 19051. (CCDC No. 1486408).

Crystal data for **(+)-3**: colorless cube crystals, $C_{15}H_{18}O_3$; M = 246.29, space group monoclinic, *C2*; unit cell dimensions were determined to be a = 19.809 (3) Å, b = 6.1007 (5) Å, c = 12.9515 (19) Å, α = 90.00 °, β = 124.46 (2) °, γ = 90.00 °, V = 1290.5 (4) Å^3, Z = 4, Dx = 1.268 mg/m^3, F (000) = 528, μ (Cu Kα) = 0.704 mm^{-1}. The final indices R_1 = 0.0335, wR_2 = 0.0838. Reflections collected: 4261. Flack parameter = 0.01 (13). (CCDC No. 1486411).

Crystal data for **(-)-3**: colorless cube crystals, $C_{15}H_{18}O_3$; M = 246.29, space group monoclinic, *C2*; unit cell dimensions were determined to be a = 19.816 (3) Å, b = 6.0941 (4) Å, c = 12.9590 (19) Å, α = 90.00 °, β = 124.40 (2) °, γ = 90.00 °, V = 1291.3 (4) Å^3, Z = 4, Dx = 1.267 mg/m^3, F (000) = 528, μ (Cu Kα) = 0.704 mm^{-1}. The final indices R_1 = 0.0439, wR_2 = 0.1080. Reflections collected: 3803. Flack parameter = 0.1 (2). (CCDC No. 1486410).

Crystal data for **5**: colorless cube crystals, $C_{15}H_{18}O_4$; M = 262.29, space group orthorhombic, $P_1\ n_1$; unit cell dimensions were determined to be a = 7.3694 (3) Å, b = 10.2747 (4) Å, c = 8.8756 (4) Å, α = 90.00 °, β = 98.066 (4) °, γ = 90.00 °, V = 665.40 (5) $Å^3$, Z = 2, Dx = 1.309 mg/m^3, F (000) = 280, μ (Cu Kα) = 0.774 mm^{-1}. The final indices R_1 = 0.0299, wR_2 = 0.0771. Reflections collected: 1274. (CCDC No. 1486409).

Quantum chemical ECD calculation of 1-4

Conformer databases of **1-3** were generated using the MMFF94S [1] force field. Conformers (ΔE 0~10.35 kJ/mol) conducted for optimizations were obtained by using density functional theory (DFT) at the B3LYP/6-311+G(d) [2] level in the gas phase based on the well summarized reports [3-8]. All the B3LYP/6-311+G(d)-optimized conformers with relative energy of 0~2.5 kcal/mol were found and used for ECD [9,10] calculations using time–dependent DFT (TDDFT) at the B3LYP/6-311++G(2d,p) [2] level.

Conformer databases of **4** were generated in CONFLEX version 7.0 using the MMFF94s force-field, with an energy window for acceptable conformers (ewindow) of 5 kcal mol^{-1} above the ground state, a maximum number of conformations per molecule (maxconfs) of 100, and an RMSD cutoff (rmsd) of 0.5Å. Then each conformer of the acceptable conformers was optimized with HF/6-31G(d) method in Gaussian09 [11]. Further optimization at the APFD/6-31G(d) level led the dihedral angles to be got. After that, eight lowest energy conformers were found out. The optimized conformers were taken for the ECD calculations, which were performed with Gaussian09 (APFD/6-311++G(2d,p)). The solvent effect was taken into account by the polarizable-conductor calculation model (IEFPCM, methanol as the solvent).

Comparisons of the experimental and calculated spectra were done with the software SpecDis [12,13]. It was also used to apply a UV shift to the ECD spectra, Gaussian broadening of the excitations, and Boltzmann weighting of the spectra.

Furthermore, OR computations [14,15] were then carried out at the same level in the gas phase. The simulated OR values were ＋167.8 for (8*S*)-**2**, －170.3 for (8*R*)-**2**. These confirm that (+)-**2** should have (*S*) absolute configuration and (-)-**2** have (*R*) absolute configuration.

NO production bioassay

Mouse monocyte-macrophage RAW 264.7 cells (ATCC TIB-71) were purchased from the Chinese Academy of Sciences. RPMI 1640 medium, penicillin, streptomycin, and fetal bovine serum were purchased from Invitrogen (New York, NY, USA). LPS, dimethylsulfoxide (DMSO), MTT, and hydrocortisone were obtained from Sigma Co. RAW 264.7 cells were suspended in RPMI 1640 medium supplemented with penicillin (100 U/mL), streptomycin (100 mg/mL), and 10% heat-inactivated fetal bovine serum. The cells were harvested with trypsin and diluted to a suspension in fresh medium. DMSO was used as a solvent for the test compounds, which were applied at a final concentration of 0.2% (v/v) in cell culture supernatants. The nitrite concentration in the medium was measured as an indicator of NO production according to the Griess reaction. Briefly, RAW 264.7 cells were seeded into 96-well tissue culture plates at a density of 1×10^5 cells per well and allowed to adhere for 2 h at 37 ℃ in a humidified atmosphere with 5% CO_2. Then, the cells were treated with 1 μg/mL of LPS in the presence or absence of test compounds. After incubation at 37 °C for 24 h, 100 μL of cell-free supernatant was mixed with 100 μL of Griess reagent (a mixture of equal volumes of reagent A and reagent B, A: 1% (w/v) sulfanilamide in 5% (w/v) phosphoric acid, B: 0.1% (w/v) of N-(1-naphthyl)-ethylenediamine. Cytotoxicity was determined by the MTT colorimetric assay after 24 h incubation with test compounds. Nitrite concentrations and the inhibitory rates were calculated using a calibration curve prepared with sodium nitrite standards.

References

1. Halgren, T. A. MMFF VI. MMFF94s option for energy minimization studies. *Journal of Computational Chemistry* **20**, 720-729 (1999).
2. Baker, J. Techniques for geometry optimization: A comparison of cartesian and natural internal coordinates. *Journal of Computational Chemistry* **14**, 1085-1100 (1993).
3. Johnson, B. G. & Frisch, M. J. Analytic second derivatives of the gradient-corrected density functional energy. Effect of quadrature weight derivatives. *Chemical Physics Letters* **216**, 133-140 (1993).
4. Stratmann, R. E., Burant, J. C., Scuseria, G. E. & Frisch, M. J. Improving harmonic vibrational frequencies calculations in density functional theory. *The Journal of Chemical Physics* **106**, 10175-10183 (1997).
5. Becke, A. D. Density‐functional thermochemistry. IV. A new dynamical correlation functional and implications for exact‐exchange mixing. *The Journal of Chemical Physics* **104**, 1040-1046 (1996).
6. Wodrich, M. D., Corminboeuf, C., Schreiner, P. R., Fokin, A. A. & von Rague Schleyer, P. How accurate are DFT treatments of organic energies? *Organic letters* **9**, 1851-1854 (2007).
7. Andzelm, J. & Wimmer, E. Density functional Gaussian‐type‐orbital approach to molecular geometries, vibrations,

and reaction energies. *The Journal of Chemical Physics* **96**, 1280-1303 (1992).
8. Zhu, H.-J. *Organic Stereochemistry: Experimental and Computational Methods*. (Wiley-VCH, 2015).
9. Yu, H. *et al.* Pestalotiopsin C, stereochemistry of a new caryophyllene from a fungus of Trichoderma sp. and its tautomerization characteristics in solution. *Tetrahedron* **71**, 3491-3494 (2015).
10. Zhang, S., Hu, D.-B., He, J.-B., Guan, K.-Y. & Zhu, H.-J. A novel tetrahydroquinoline acid and a new racemic benzofuranone from Capparis spinosa L., a case study of absolute configuration determination using quantum methods. *Tetrahedron* **70**, 869-873 (2014).
11. Frisch, M.J., et al. Gaussian, Inc., Wallingford CT, 2010.
12. Bruhn, T., Schaumlöffel, A., Hemberger, Y. & Bringmann, G.; Version 1.61 ed.; University of Würzburg: Würzburg, Germany, 2013.
13. Bruhn, T., Schaumloffel, A., Hemberger, Y. & Bringmann, G. SpecDis: quantifying the comparison of calculated and experimental electronic circular dichroism spectra. *Chirality* **25**, 243-249 (2013).
14. Liao, T.-G., Ren, J., Fan, H.-F., Xie, M.-J. & Zhu, H.-J. Study of syntheses and specific rotations of (S)-3-phenylhexan-3-ol and its derivatives. *Tetrahedron: Asymmetry* **19**, 808-815 (2008).
15. Zhao, S.-D., Shen, L., Luo, D.-Q. & Zhu, H.-J. Progression of Absolute Configuration Determination in Natural Product Chemistry Using Optical Rotation (Dispersion), Matrix Determinant and Electronic Circular Dichroism Methods. *Current Organic Chemistry* **15**, 1843-1862 (2011).
